# Advancing Personalized Medicine in Alzheimer’s Disease: Liquid Biopsy Epigenomics Unveil *APOE* ε4-Linked Methylation Signatures

**DOI:** 10.3390/ijms26073419

**Published:** 2025-04-05

**Authors:** Mónica Macías, Juan José Alba-Linares, Blanca Acha, Idoia Blanco-Luquin, Agustín F. Fernández, Johana Álvarez-Jiménez, Amaya Urdánoz-Casado, Miren Roldan, Maitane Robles, Eneko Cabezon-Arteta, Daniel Alcolea, Javier Sánchez Ruiz de Gordoa, Jon Corroza, Carolina Cabello, María Elena Erro, Ivonne Jericó, Mario F. Fraga, Maite Mendioroz

**Affiliations:** 1 Neuroepigenetics Unit, Navarrabiomed, Hospital Universitario de Navarra, Universidad Pública de Navarra, Navarra Institute for Health Research (IdiSNA), 31008 Pamplona, Spain; 2 Cancer Epigenetics and Nanomedicine Laboratory, Nanomaterials and Nanotechnology Research Center (CINN CSIC), 33940 El Entrego, Spain; 3 Health Research Institute of Asturias (ISPA FINBA), University of Oviedo, 33011 Oviedo, Spain; 4 Institute of Oncology of Asturias (IUOPA), University of Oviedo, 33006 Oviedo, Spain; 5 Rare Diseases CIBER (CIBERER) of the Carlos III Health Institute (ISCIII), 28029 Madrid, Spain; 6 Department of Neurology, Institut d’Investigacions Biomèdiques Sant Pau (IIB Sant Pau), Hospital de la Santa Creu i Sant Pau, Universitat Autònoma de Barcelona, 08025 Barcelona, Spain; 7 Centro de Investigación Biomédica en Red en Enfermedades Neurodegenerativas, CIBERNED, 28029 Madrid, Spain; 8 Neurology Department, Hospital Universitario de Navarra, Universidad Pública de Navarra, Navarra Institute for Health Research (IdiSNA), 31008 Pamplona, Spain; 9 Department of Organisms and Systems Biology (B.O.S.), University of Oviedo, 33006 Oviedo, Spain

**Keywords:** Alzheimer’s disease, cell-free DNA, liquid biopsy, EPIC array, DNA methylation, *APOE* ε4, blood

## Abstract

Recent studies show that patients with Alzheimer’s disease (AD) harbor specific methylation marks in the brain that, if accessible, could be used as epigenetic biomarkers. Liquid biopsy enables the study of circulating cell-free DNA (cfDNA) fragments originated from dead cells, including neurons affected by neurodegenerative processes. Here, we isolated and epigenetically characterized plasma cfDNA from 35 patients with AD and 35 cognitively healthy controls by using the Infinium^®^ MethylationEPIC BeadChip array. Bioinformatics analysis was performed to identify differential methylation positions (DMPs) and regions (DMRs), including *APOE* ε4 genotype stratified analysis. Plasma pTau181 (Simoa) and cerebrospinal fluid (CSF) core biomarkers (Fujirebio) were also measured and correlated with differential methylation marks. Validation was performed with bisulfite pyrosequencing and bisulfite cloning sequencing. Epigenome-wide cfDNA analysis identified 102 DMPs associated with AD status. Most DMPs correlated with clinical cognitive and functional tests including 60% for Mini-Mental State Examination (MMSE) and 80% for Global Deterioration Scale (GDS), and with AD blood and CSF biomarkers. In silico functional analysis connected 30 DMPs to neurological processes, identifying key regulators such as *SPTBN4* and *APOE* genes. Several DMRs were annotated to genes previously reported to harbor epigenetic brain changes in AD (*HKR1*, *ZNF154*, *HOXA5*, *TRIM40*, *ATG16L2*, *ADAMST2*) and were linked to *APOE* ε4 genotypes. Notably, a DMR in the *HKR1* gene, previously shown to be hypermethylated in the AD hippocampus, was validated in cfDNA from an orthogonal perspective. These results support the feasibility of studying cfDNA to identify potential epigenetic biomarkers in AD. Thus, liquid biopsy could improve non-invasive AD diagnosis and aid personalized medicine by detecting epigenetic brain markers in blood.

## 1. Introduction

Alzheimer’s disease (AD) represents the primary cause of age-related dementia and the seventh leading cause of mortality globally [1]. The majority of AD cases occur sporadically in adults older than 65 years, referred to as late-onset AD (LOAD). With the ever increasing aging of the population, this neurodegenerative disease currently affects 1 in 9 people over the age of 65, and its prevalence is projected to reach 152 million people worldwide by 2050 [2,3]. Despite its significant impact, the underlying mechanisms for AD pathogenesis remain unclear. Enhancing the accuracy of AD diagnosis would optimize early therapeutic intervention strategies, thereby reducing costs and the increasing burden that AD represents for our society.

Multiple factors, such as biological, environmental, and genetic susceptibility, appear to be associated with the development of LOAD. Within genetic factors, *APOE* ε4 polymorphism has been found to be the most consistently associated with LOAD development [4]. In recent years, epigenetics has emerged as a significant player in the pathogenesis of neurodegenerative diseases such as AD [5]. Among different epigenetic modifications, DNA methylation—where a methyl group attaches to the 5-carbon position of a cytosine base, typically in cytosine guanine dinucleotides (CpGs)—has been extensively studied. In the case of AD, gene candidate studies, along with the latest application of omics technologies to epigenetics, have revealed new DNA methylation variants in genes biologically relevant to AD in human brain tissue.

Our group and others have published epigenome-wide studies describing differentially methylated genes in various brain regions using postmortem human samples. These regions include the prefrontal cortex [6,7,8,9,10,11,12,13,14], frontal cortex [15], entorhinal cortex [9,10,11,14], hippocampus [14,16], or superior temporal gyrus and inferior frontal gyrus [11,13,17,18]. However, a major obstacle hinders the translation of these promising findings as biomarkers to clinical practice: the difficulty of accessing brain tissue from living individuals with AD. As a result, the AD specific epigenetic information remains “trapped” within the brain tissue and, therefore, rather inaccessible while the patient is alive. Studies performed on blood-derived genomic DNA have also identified differentially methylated marks between AD patients and controls [19,20,21,22]. Nonetheless, most of these marks do not match those observed in brain tissues.

It is widely acknowledged that cells undergo necrosis and apoptosis, among various processes of cell death, leading to the release of their DNA into the bloodstream. This DNA, characterized by specific molecular alterations, is commonly referred to as cell-free DNA (cfDNA). Liquid biopsy is a non-invasive technique involving a blood test that enables the isolation of cfDNA from plasma [23]. Under normal conditions, cfDNA primarily originates from the apoptosis of peripheral white blood cells [24]. However, as evidenced by the enrichment of tissue-specific methylation marks, a considerable proportion of cfDNA originates from damaged tissues during pathological processes [25].

To date, most liquid biopsy applications have mainly concentrated on the identification of genetic variants, such as tumor specific alterations. Nevertheless, liquid biopsy is emerging as a valuable tool in neurodegenerative diseases [26,27,28,29] where: (i) the blood–brain barrier is dysfunctional, increasing its permeability [30,31]; and (ii) there are no genetic modifications on the DNA of the affected cells. In these diseases, the analysis of epigenetic modifications in cfDNA specimens arises as a novel source of diagnostic biomarker. Variants in DNA methylation, in particular, are considered outstanding biomarkers because of their stability, potential reversibility, and accessibility in body fluids [32].

The liquid biopsy technique would provide access to this information “trapped” in the brain, enabling the identification of epigenetic biomarkers (specific methylation marks) in cfDNA from patients with AD. This molecular assessment of cfDNA specimens could be thus considered a potential surrogate for pathological studies of postmortem brain tissue, offering a potential source of epigenetic biomarkers that could assist in the clinical care of AD throughout the patient’s lifetime.

Hence, the aim of this study was to identify differential methylation signatures of plasma cfDNA in patients with AD compared with controls through a genome-wide methylation analysis.

## 2. Results

### 2.1. Characterization of Subjects and Samples

The Infinium^®^ MethylationEPIC BeadChip microarray (EPIC array) was applied to a set of 35 controls and 35 patients with AD. No significant differences in age or sex were observed between the subjects with AD and the controls. Extended demographic and clinical features of the subjects are summarized in Table 1.

### 2.2. cfDNA Concentration and Quality

We managed to isolate plasma cfDNA from all the subjects included in this study. The amounts of cfDNA did not vary significantly between the controls and the patients with AD (median: 96 ng; IQR = 47–212 vs. median: 81 ng; IQR = 34–241; *p*-value = 0.445), respectively. First, we verified the cfDNA corresponding size in our sample set as described in the methods section using the DNF 477 High Small Fragment Analysis Kit (Agilent). The expected cfDNA size was confirmed in all samples. The median cfDNA size was 167 bp (IQR = 158 185) for patients with AD and 165 bp (IQR = 159 171) for controls, with no significant differences between groups (*p*-value = 0.451). An example electropherogram of a cfDNA sample is presented in Supplementary Materials: Figure S1.

### 2.3. Surrogate Variable Analysis

Surrogate variable analysis revealed one confounding variable of unknown significance (SV1) as the major source of variability affecting our series (Supplementary Materials: Figure S2). To ascertain the nature of this biological or technical variable, we decided to further characterize the cfDNA fragmentation pattern employing fragment analyzer technology. We found several samples with a carryover of non-sized cfDNA (Supplementary Materials: Figure S3a). To specifically quantify this cfDNA fraction and evaluate its impact, we decided to perform a more in depth characterization of the isolated cfDNA using the DNF 464 High Sensitivity Large Fragment 50 Kb Analysis Kit. This kit allows to track the cfDNA fragmentation pattern from 75 bp to 48,500 bp, covering both the expected cfDNA expected fragment size and an extended region with other potential non cfDNA fragments.

Interestingly, we noticed that non-sized cfDNA fragments were present in several samples, thus contributing to the total concentration. An example is shown in Supplementary Materials: Figure S3b. We observed that SV1 was negatively correlated with non-sized cfDNA. Global evaluation of non-sized cfDNA revealed a presence of 42.24% in controls and 54.89% in patients with AD, with no significant differences between groups (*p*-value = 0.07) (Supplementary Materials: Figure S4). Nevertheless, upon further examination of the influence of non-sized cfDNA on methylation levels, we observed a strong positive correlation between non-sized cfDNA and median β methylation values (r = 0.445; *p*-value < 0.001). Therefore, the non-sized cfDNA percentage was used to adjust all the subsequent analyses.

### 2.4. Differential Methylated Positions

After quality control and sample tracking, seven samples were discarded from downstream analysis. Finally, cfDNA from 30 controls and 33 patients with AD was used for differential methylation analysis. We confirmed that the loss of these subjects did not result in any changes leading to differences between patients with AD and controls in terms of phenotypical features, as illustrated in Supplementary Materials: Table S1. Genome-wide DNA methylation was investigated in the context of both differentially methylated positions (DMPs) and differentially methylated regions (DMRs). First, we built mixed linear models, adjusting for potential sources of variability, specifically including sex, age, batch, non-sized cfDNA (%), and cell type composition. After adjusting for Benjamini–Hochberg method (FDR) correction, we detected no significant DMPs associated with AD condition. When looking at the nominal significance level, analysis revealed 102 AD-related DMPs (absolute β difference ≥ 0.1 and *p*-value ≤ 0.05) annotated to 58 genes (Table 2), with an overrepresentation of hypomethylated positions in AD cases compared with controls (74%).

**Figure 1:**
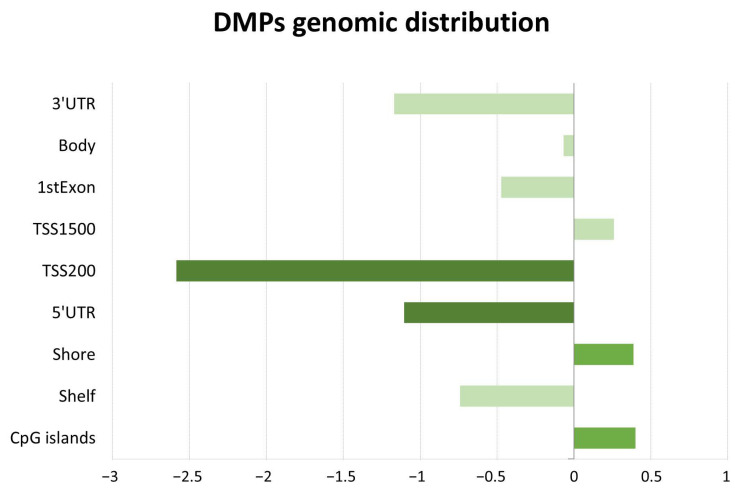
Gene structure distribution of DMPs between patients with Alzheimer’s disease (AD) and controls. The bar chart shows results for the log2 ratios of observed (fraction of differentially methylated probes overlapping a given region) to expected (fraction of probes selected for analysis overlapping a given region) for a genomic region. Dark green boxes represent a significant enrichment (*p*-value < 0.05) for a particular feature. TSS = number of nucleotides upstream and downstream of the transcription start site.

The genomic distribution of AD-related DMPs was assessed for differential enrichment in terms of CpG context and genomic regions (Figure 1). We observed that DMPs were more frequently located in CpG islands and shore regions, exhibiting a 1.3-fold enrichment (*p*-value < 0.05) compared with the random expectation based on all probes included in the analysis.

### 2.5. Correlation with AD Clinical Parameters and Biomarkers

Subsequently, Spearman’s coefficient was calculated to evaluate the potential correlation between DNA methylation levels of the top ten DMPs, ranked by the highest positive and negative β difference criteria, respectively, and main AD clinical features and biomarkers. Very interestingly, we found significant correlations between DNA methylation levels of DMPs and MMSE score in 12/20 (60%), and with GDS in 16/20 (80%) (Table 3 and Table 4).

Regarding biomarkers, we observed a significant correlation with the CSF Aβ42/Aβ40 ratio in 2/20 (10%) and with plasma pTau181 concentration in 7/20 (35%) (Table 3 and Table 4). We also observed several correlation trends, although without reaching significance for CSF pTau181 in up to 6/20 (30%). Interestingly, three genes significantly correlated with MMSE, GDS, and pTau181 levels, namely *SMTNL2*, *GLRA1*, and *MORC2-AS1*.

### 2.6. Functional in Silico Analysis of DMPs

We conducted ingenuity pathway analysis (IPA) to further explore the biological significance of AD-related DMPs identified in this study. Within the “diseases and functions” category, the analysis revealed that up to 30 molecules among our set of AD-related DMPs were associated with neurological disorders (*p*-value range = 4.53 × 10^−2^–1.10 × 10^−3^) (Supplementary Materials: Table S2). In the “physiological system development and function” category, we found that 11 molecules in our dataset were primarily mostly enriched in nervous system development and function (*p*-value range = 4.95 × 10^−2^–2.83× 10^−5^) (Supplementary Materials: Table S2).

**Figure 2:**
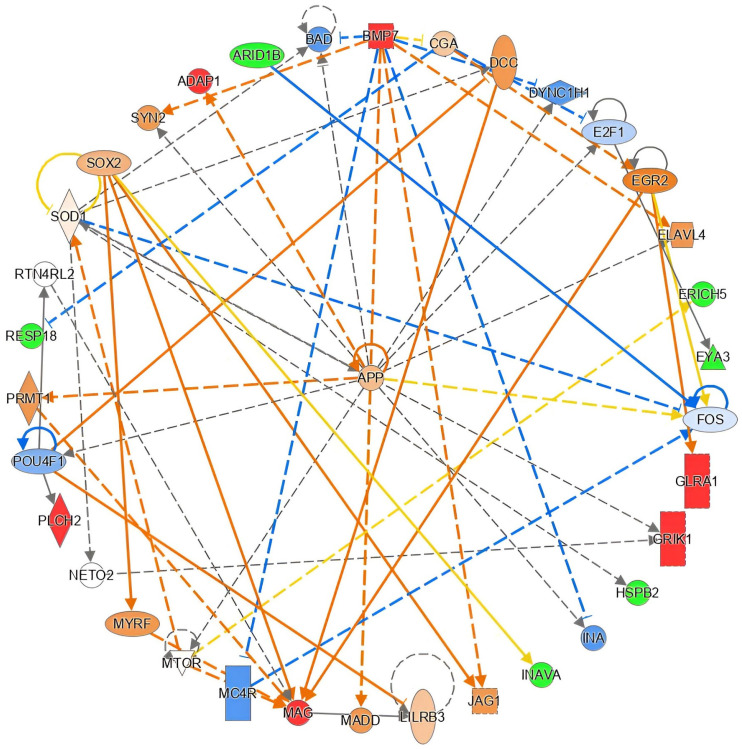
*APP* gene plays a central role in the principal network evolving our AD-related DMPs. The graph shows how amyloid precursor protein-encoding gene (*APP*) acts as a core regulator of 12 genes found in our dataset (IPA score = 23). Red and green coloring indicate increased/decreased measurement in our dataset, respectively. Orange and blue coloring indicate predicted activation/inhibition genes participating. The intensity of the color refers to the strength of the effect.

Furthermore, IPA analysis predicted 24 upstream transcriptional regulators directly or indirectly linked to the genes in our dataset, prioritized by *p*-value. Among these, *SPTBN4* (spectrin β non-erythrocytic 4), which encodes a brain cytoskeletal protein, emerged as the most significantly associated regulator (Supplementary Material: Table S3). Also remarkable was the presence of the *APOE* ε4 gene among these upstream regulators. Moreover, causal network analysis (CNA) [33] further connected upstream regulators to our dataset molecules, placing the *APP* gene (amyloid β precursor protein), which encodes a membrane protein mainly expressed in neuronal synapses and closely related to AD development, at the forefront of potential relationships (Figure 2).

### 2.7. Differential Methylated Positions According to APOE ε4 Status

As previously mentioned, the *APOE* ε4 genotype is considered the strongest genetic risk factor for LOAD, and DNA methylation has demonstrated to act closely with this factor, revealing DNA methylation differences between *APOE* ε4 carriers and non-carriers [34]. Therefore, to explore our results in greater depth, we divided our sample set regarding *APOE* ε4 status in each group of subjects as follows: patients with AD *APOE* ε4 carriers (*n* = 19; 58%), patients with AD *APOE* ε4 non-carriers (*n* = 14; 42%), control *APOE* ε4 carriers (*n* = 3; 10%), and control *APOE* ε4 non-carriers (*n* = 27; 90%). No comparison was performed with control *APOE* ε4 carriers due to the minimum number of *APOE* ε4 carriers in the control group.

When looking at the nominal significance level, major differences were found when comparing AD *APOE* ε4 carriers and control *APOE* ε4 non-carriers, represented by 980 DMPs (absolute β difference ≥ 0.1 and *p*-value ≤ 0.05) annotated to 668 genes (Supplementary Materials: Table S4). When comparing AD *APOE* ε4 non-carriers and control *APOE* ε4 non-carriers, we found 286 DMPs (absolute β difference ≥ 0.1 and *p*-value ≤ 0.05) annotated to 169 genes (Supplementary Materials: Table S5).

### 2.8. Differential Methylated Regions

At a regional level, differential analysis revealed one DMR significantly associated with AD status (Sidak corrected *p*-value < 0.05). This position (chr2:114,737,458–114,737,475) was located in a CpG island and annotated to a lncRNA (LOC100499194).

When stratifying by *APOE* ε4, we identified 17 DMRs (12% hypermethylated and 88% hypomethylated) comparing AD *APOE* ε4 carriers and control *APOE* ε4 non-carriers and 4 hypermethylated DMRs between AD *APOE* ε4 non-carriers and control *APOE* ε4 non-carriers (Sidak correction *p*-value < 0.05) (Table 5 and Table 6). Most interestingly, up to six DMRs were annotated to genes already addressed as differentially methylated in AD condition and mostly in brain tissue (Table 7).

### 2.9. Orthogonal Validation

**Figure 3:**
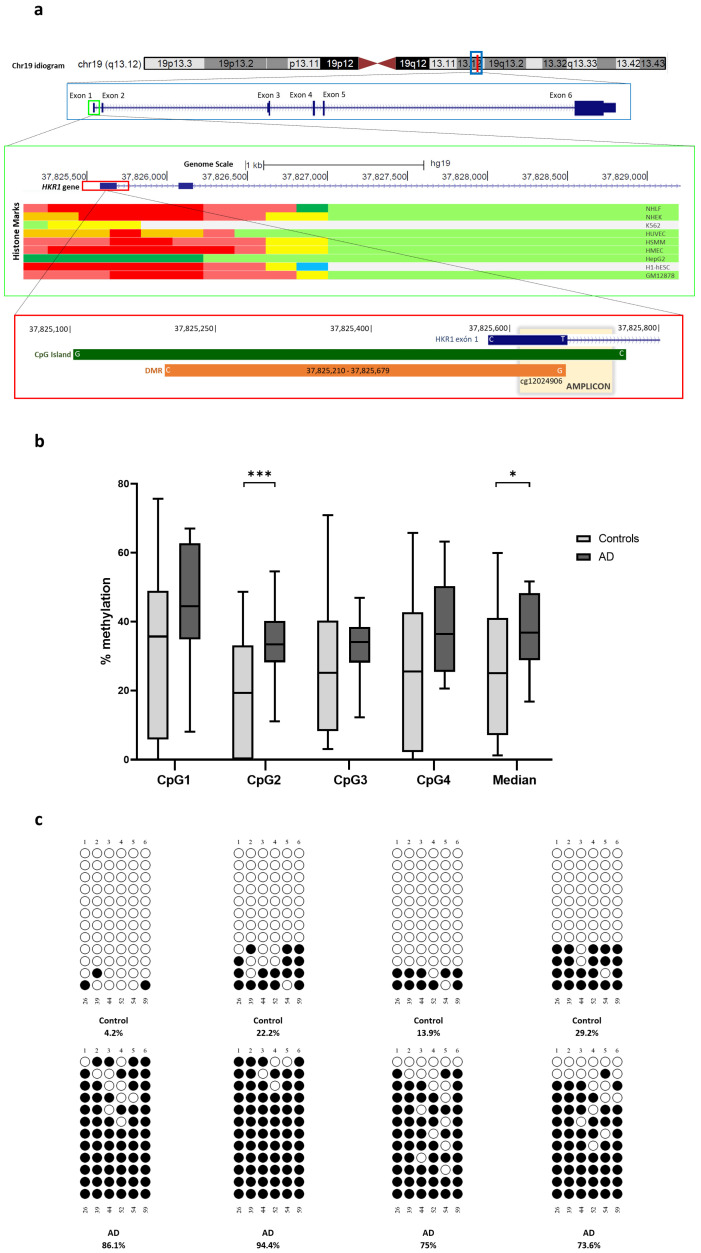
Differential methylated region (DMR) annotated to *HKR1* gene in plasma cfDNA from Alzheimer’s disease (AD) and control subjects. (**a**) The graph depicts the genomic location of the amplicon covering the DMR within the *HKR1* gene’s promoter region analyzed by bisulfite cloning sequencing. Functional elements predicted for nine human cell lines, identified through chromatin immunoprecipitation (ChIP) combined with massively parallel DNA sequencing, are displayed in the middle of the graph. The track was obtained from chromatin state segmentation by HMM from ENCODE/Broad track, shown in the UCSC Genome Browser. At the bottom, the CpG island is represented by a green box, the DMR by an orange box, and the amplicon spanning the DMR is represented in yellow. (**b**) *HKR1* cfDNA methylation levels measured by pyrosequencing. Box plot charts display the methylation levels for individual CpG sites within the amplicon and the average levels between patients with Alzheimer’s disease (AD) and controls. Horizontal lines represent median methylation values and interquartile range for each group. * *p*-value < 0.05; *** *p*-value < 0.001 (Student’s *t* test). (**c**) Representative examples of bisulfite cloning sequencing validation for the amplicon containing the CpG are shown. Black and white circles denote methylated and unmethylated cytosines, respectively. Each column symbolizes a unique CpG site in the examined amplicon and each line represents an individual DNA clone. CpG—cytosine guanine dinucleotide.

A DMR found between AD *APOE* ε4 non-carriers and control non-carriers annotated to the *HKR1* gene (*ZNF875*, zinc finger protein 875), a gene previously found to be differentially methylated in the hippocampus, was selected for further exploration (Figure 3a). This DMR consists of 469 bp containing 10 CpG dinucleotides. Considering that the expected cfDNA size is around 166 bp and further fragmentation likely occurs during the deamination step of the bisulfite conversion process, special caution was exercised in designing the region to be explored [35]. For primer design, we selected a CpG assayed in the EPIC array (cg12024906) located in the extreme of the DMR. Therefore, we designed primers to achieve amplicons contained in the DMR smaller than the estimated cfDNA size.

For pyrosequencing, we examined a 140 bp region covering four CpG dinucleotides, including cg12024906. We observed that DNA methylations levels at the selected CpG site (CpG2) and the average for the amplicon were significantly increased in patients with AD compared with the controls [30.74 ± 14.57% vs. 18.92 ± 16.41%, *p*-value < 0.01 and 36.51 ± 11.85% vs. 26.02 ± 19.48%, *p*-value < 0.05, respectively) (Figure 3b).

For bisulfite cloning sequencing, we analyzed an 86 bp region encompassing six CpG dinucleotides in eight representative samples. We observed that the average DNA methylation levels were strongly higher in patients with AD compared with the controls, both for the amplicon [82.28 ± 9.83% vs. 17.36 ± 10.78%; *p*-value < 0.001] and the selected CpG site (CpG4) [62.50 ± 17.33% vs. 14.58 ± 14.22%; *p*-value < 0.001] (Figure 3c).

Overall results of this orthogonal validation are detailed in Supplementary Materials: Table S6.

## 3. Discussion

The aim of this study was to identify differential methylation signatures in plasma cfDNA as a potential non-invasive source of epigenetic biomarkers for patients with AD. We hereby demonstrate that cfDNA can be efficiently isolated from plasma through liquid biopsy procedures and used to identify methylation differences between patients with AD and cognitively healthy controls. Specific cfDNA methylation differences seem to be *APOE* ε4 genotype-related. In particular, four DMRs were found in *APOE* ε4 non-carriers when comparing AD versus control subjects.

Methodologies to analyze cfDNA in biological fluids are greatly technologically demanding in terms of sensitivity, given the low levels of cfDNA (typically around 10 ng/mL). In neurological disorders, this challenge is compounded by the low relative abundance of the expected brain-derived fraction among the background cfDNA [27]. A way to achieve higher starting amounts of cfDNA could be by drawing larger volumes of plasma; however, there should be a plasma volume limitation for this technique to be transferable to neurological clinical practice in the near future, if differences in DNA methylation are intended to be used as biomarkers. Another approach could involve sample pooling techniques to increase the available starting material when using cfDNA, as previously suggested [36]. However, this approach only yields average methylation values. Additionally, cfDNA appears at higher levels in certain pathological conditions, such as cancer, trauma, stroke, autoimmune disorders, or insufficient renal clearance [37]. In this regard, we used as an exclusion criterion, for both controls and patients with AD, the co-existence of another pathological condition that could potentially overestimate plasma AD-related cfDNA concentrations. In our study cohort, we obtained cfDNA from the plasma of all patients with AD and the controls. Nevertheless, we did not find a significant increase in total plasma cfDNA levels in patients with AD compared with the controls in our cohort, which adds evidence in this regard, since previous studies showed conflicting results in terms of these differences [38,39]. This could be explained by the low relative abundance of the expected brain-derived cfDNA within the background cfDNA, making it challenging to detect any increase in the concentration of the brain-derived fraction in the total cfDNA.

A critical aspect for cfDNA analysis involves preanalytical factors, such as the type of collection tube, sample centrifugation protocol, and cfDNA extraction method [40]. Although commercial kit manufacturers usually claim efficient isolation of pure high quality cfDNA, this study highlights the significant impact of non-sized cfDNA carryover [41,42]. Non-sized cfDNA contamination often goes unnoticed when employing common cfDNA quantification methods, such as fluorometric techniques, which cannot distinguish between cfDNA and genomic DNA (gDNA) [42]. Our findings indicate that approximately half of the samples contained non-sized cfDNA carryover. Nevertheless, the presence of non-sized cfDNA did not significantly differ between patients with AD and the controls in our study cohort. Given that non-sized cfDNA was identified as a major source of variability in the surrogate variable analysis, we decided to adjust our statistical models to account for this variable as a latent source of noise.

Several research groups have conducted epigenome-wide studies on cfDNA from patients with AD using various methods, including targeted bisulfite sequencing [43], high throughput sequencing to map 5-hydroxymethylcytosine (5hmC) as another widely used epigenetic marker [44], and even EPIC array technology [45,46]. In recent years, the Illumina Infinium^®^ MethylationEPIC BeadChip array has emerged as a widely used and accessible option. Similar to whole genome bisulfite sequencing (WGBS), this technology utilizes sodium bisulfite DNA conversion, followed by single-base resolution genotyping of specific CpG sites through microarray probes. The EPIC platform stands out for its efficiency, affordability, and consistency with DNA methylation results obtained from other methods [47].

In our cohort, and using this microarray technology, we found no significant DMPs associated with AD condition in cfDNA after applying a FDR < 0.05 correction. This finding aligns with previous epigenome studies performed on cfDNA [44,48]. Other than technical factors and analysis methodologies, bioinformatics filtering thresholds could represent a major source of discordance between assays. A recent paper by Bahado Singh et al. [45] reported significant differences in cfDNA between patients with AD and controls after FDR correction employing EPIC array technology and artificial intelligence algorithms for data analysis. However, in their study, only about 41% of the total probes assayed by the EPIC array passed quality control, whereas in our study, up to 86% of the overall probes were included in the subsequent differential methylation analysis. Another strategy employed by the same group was to focus bioinformatic data analysis on a specific subset of CpGs among all methylation sites across the genome, in this case, the methylation in cytochrome p450 (CYP) genes [46].

However, the analysis identified an interesting set of 102 differential cfDNA methylation marks at a nominal significance level. Among these methylation marks, hypomethylation in AD cases compared with controls was overrepresented. Regarding genomic location, these differential cfDNA methylation marks were predominantly localized in CpG islands and shores, concordant with previous findings in genomic DNA in patients with AD [17]. Moreover, we found strong correlations between the ten top-ranked nominally significant probes in our dataset and main cognitive and functional status indicators (MMSE and GDS), along with mild correlations with AD biomarkers in CSF and blood, such as Aβ42/Aβ40 ratio and pTau181 levels, respectively.

In the functional interpretation of results, IPA provides a comprehensive visualization of how a gene dataset impacts a pathway, presenting it as a network to enhance the understanding of the findings. The most significant upstream regulator identified the *SPTBN4* gene, with *ANK3* being its main target molecule in our dataset. *SPTBN4* encodes for spectrin β non-erythrocytic 4 and, along with ANK3, a member of the ankyrin family, forms part of the axon initial segment [49]. The spectrin/ankyrin complex links the cytoskeleton and voltage gated channels, which are crucial for regulating neuronal polarity and synapsis [50]. Interestingly, Sánchez Mut et al. reported hypermethylation of *SPTBN4* in the frontal cortex of human patients with AD [51]. Also remarkable is the presence of *APOE* ε4 among this gene list, interacting again with *ANK3* and with *ADGRB1* (adhesion G protein-coupled receptor B1, brain-specific angiogenesis inhibitor, *BAI1*), which mediates hippocampal spatial learning and memory [52].

To the best of our knowledge, this is the first *APOE* ε4-stratified study conducted with the Infinium EPIC array on cfDNA obtained from patients with AD. The *APOE* ε4 genotype is widely recognized as an important risk factor for AD and may affect the progression of the disease, potentially influencing the status of AD-associated DNA methylation marks [21]. Therefore, we decided to further include the *APOE* ε4 genotype into our linear models to analyze its contribution to the cfDNA methylation differences observed in this study. When stratifying patients with AD and controls based on the presence of the *APOE* ε4 genotype, we observed a substantial increase in differentially methylated positions by 10%. This finding aligns with previous methylation studies that have demonstrated clear and significant different methylation marks between patients with AD and controls when stratifying by the *APOE* ε4 genotype [34,53,54]. However, further research is required to clarify the mechanisms that connect DNA methylation with the presence of the ε4 allele.

Nevertheless, the most significant differences were found when exploring results at a regional level, probably because position level differences are often too subtle to be detectable [19]. In this regard, we identified several cfDNA methylation differences in regions associated with genes known to be important in AD. For instance, we found a hypermethylated region in AD *APOE* ε4 non-carriers annotated to the *SLCO2A1* gene (solute carrier organic anion transporter family member 2A1). This gene encodes for prostaglandin transporter, which is reported to be localized in neurons, microglia, and astrocytes, and is poorly expressed in the AD human brain. This transporter has been suggested as a possible modulator of prostaglandin-driven neuroinflammation linked to AD [55]. In addition, we detected a hypomethylated region in AD *APOE* ε4 carriers spanning the *DNMT3B* gene, which encodes DNA methyltransferase 3 Β, an enzyme that catalyzes de novo DNA methylation in mammalian cells [56]. It would be interesting to check whether *DNMT3B* hypomethylation identified in AD *APOE* ε4 carriers may be related to the higher expression of these DNA methyltransferases with increasing age [57]. This deregulation in DNMT-mediated de novo methylation due to its own methylation state may help to explain the AD epigenetic misbalance [58]. Notably, we identified this DMR when comparing AD *APOE* ε4 carriers with control *APOE* ε4 non-carriers. The association between *DNMT3B* deregulation and the *APOE* ε4 genotype has been previously addressed, indicating synergistic effects on AD onset [59]. However, studies elsewhere have found no significant correlation between *DNMT3B* methylation and *APOE* ε4 status [60]. These findings within our dataset may help to uncover new underlying molecular changes associated with AD pathology.

Liquid biopsy has emerged as a non-invasive tool for reflecting molecular changes occurring in tissues. Interestingly, we observed how some of the AD-related regional changes identified in plasma cfDNA in our study cohort were consistent with changes previously reported in other studies performed on brain samples (Table 7). In particular, several DMRs identified in this study were associated with genes previously reported as differentially methylated in the hippocampus of patients with AD, such as *HKR1* and *ATG16L2*. The *HKR1* gene (*ZNF875*, zinc finger protein 875) gene is a transcriptional regulator, and its methylation levels have been proposed as an aging biomarker [61]. In this study, we observed that *HKR1* methylation changes remained significant in patients with AD after adjusting for age, which may indicate underlying age-related epigenetic changes contributing to neurodegeneration in AD [62,63]. Moreover, both *HKR1* and *ATG16L2* methylation levels were found to correlate significantly with pTau burden in the AD hippocampus [16]. In this respect, we found no correlation between cfDNA methylation levels of these DMRs and tTau/pTau181 and pTau181 as surrogate biomarkers for Tau deposition in CSF and blood, respectively.

Our study adds evidence to using cfDNA to characterize methylation changes in neurological diseases, such as AD. Plasma cfDNA emerges as a novel source of epigenetic biomarker that may help to improve AD diagnosis in the clinical setting. Precision medicine based on liquid biopsy procedures is an innovative approach that merits further research. Validation of these candidate biomarkers in larger and multicentric cohorts will contribute to the design of panels of composite biomarkers from different origins that may significantly improve clinical tools in the dementia field. In this respect, ultra-sensitive technologies such as droplet digital PCR (ddPCR) have great potential for the validation of these candidate biomarkers in plasma cfDNA [64].

### Limitations

The primary limitations of our study are those related to the nature of the cfDNA. In this work we have demonstrated that non-sized cfDNA carryover in standard cfDNA isolation protocols can go unnoticed, which should be considered when analyzing results [65]. A possible approach to overcome non-sized cfDNA carryover could be the further use of methods based on capillary electrophoresis to selectively elute DNA according to its base pair length [66]. Future investigations should aim to validate these results across larger diverse independent cohorts to definitively establish both the clinical utility and the reproducibility of our findings.

## 4. Materials and Methods

### 4.1. Study Design

We conducted an observational case control study including 70 subjects (35 patients with AD and 35 age- and sex-matched cognitively healthy controls) to identify cfDNA methylation differences between patients with AD and controls by using liquid biopsy procedures.

### 4.2. Subjects’ Characterization

Patients were prospectively enrolled from the dementia unit at the University Hospital of Navarra (a tertiary hospital) between March 2019 and December 2021. AD was diagnosed following the National Institute on Aging and Alzheimer’s Association (NIA-AA 2018) guidelines [67]. The diagnosis was made by neurologists based on the patient’s medical history, clinical examination, blood tests, neuropsychological assessments, and magnetic resonance imaging (MRI) scans. Cognitive function was evaluated by the Mini-Mental State Examination (MMSE) [68] and the Global Deterioration Scale (GDS) [69]. Healthy controls were recruited from relatives and volunteers matched for age and sex, and with no clinical signs of dementia or other neurodegenerative diseases, as confirmed through clinical interviews and MMSE (score > 27). Given that cfDNA concentrations increase in cancer stages [70], we exclusively enrolled controls and patients with AD who had not experienced any tumor disease within, at least, the last five years. This study was approved by the ethics committee, and all participants provided written informed consent prior to their involvement.

The sample size was calculated to provide 80% statistical power to detect a minimum significant difference of 10% in cfDNA methylation levels between AD cases and controls. It was assumed that both distributions followed a normal pattern with equal variance (σ = 0.15) and that an independent samples *t* test would be applied at a two-sided significance level of α = 0.05. Based on these parameters, the necessary sample size was determined to be 35 patients with AD and 35 controls, using the *epiR* library of the R statistical package (v.4.0.2) [71].

### 4.3. Blood and Cerebrospinal Fluid (CSF) Samples

Peripheral blood samples were obtained from each participant by venipuncture into 10 mL PAXgene^®^ Blood DNA Tubes (QIAGEN, Redwood City, CA, USA), which contained a leukocyte stabilizer to prevent contamination with genomic DNA. The collected samples were centrifuged at 1900× *g* at room temperature for 15 min within 1 h of collection. Plasma was then transferred to plastic tubes, subjected to a second centrifugation at maximum speed, and stored at −80 °C until further analysis. For additional analysis, pTau181 was measured in additional EDTA plasma samples from both patients with AD and controls, whenever these samples were available. This measurement was performed using the commercially available pTau181 V2 Advantage kit (Quanterix Corp, Billerica, MA, USA), with single molecule array (Simoa) technology at Sant Pau Memory Unit’s laboratory (Barcelona, Spain).

As part of their clinical diagnosis, 21 out of 35 patients with AD underwent lumbar puncture for CSF biomarker testing to further classify their amyloid/tau/neurodegeneration (ATN) profile [67]. CSF samples were collected by lumbar puncture, centrifuged at 2000× *g* for 10 min at 4 °C within 4 h after collection, and the supernatants were aliquoted into 1.5 mL polypropylene tubes. These aliquots were stored at −80 °C until further use. Aβ42, Aβ40, pTau181, and tTau in CSF were measured using a Lumipulse G600II instrument (Fujirebio, Ghent, Belgium), according to the manufacturer’s instructions.

### 4.4. cfDNA Isolation and Quantification

cfDNA was isolated from 2 mL plasma by using QIAmp Circulating Nucleic Acid Kit (QIAGEN, Redwood City, CA, USA), following the manufacturer’s protocol. The concentration of double-stranded cfDNA was quantified using a Qubit 2.0 Fluorometer with the Qubit dsDNA HS Assay Kit (Thermo Fisher Scientific, Gilford, NH, USA), as per the manufacturer’s guidelines. The amounts of cfDNA used for the methylation differential analysis array were reported in nanograms (ng).

### 4.5. Characterization of cfDNA: Fragment Size Analysis

The purity and size distribution of cfDNA fragments were analyzed using the Fragment AnalyzerTM Automated CE with ProSize software v. 3.0 (Agilent, Technologies, Inc., Santa Clara, CA, USA). This analysis was conducted with the DNF 477 HS Small Fragment kit and the DNF 464 High Sensitivity Large Fragment 50 Kb Analysis Kit (Agilent), following the manufacturer’s instructions.

### 4.6. Genome-Wide cfDNA Methylation Analysis

cfDNA from 70 plasma samples was treated with sodium bisulfite using the Zymo EZ 96 DNA methylation kit (Zymo Research, Irvine, CA, USA), following the manufacturer’s protocol. Since cfDNA is highly fragmented, we treated our sample set with the Illumina Infinium FFPE restoration kit (Illumina, San Diego, CA, USA) prior to methylation analysis to protect the samples during the bisulfite conversion process, as previously described [72].

Subsequently, a methylome analysis was conducted on cfDNA samples using the Illumina Infinium^®^ MethylationEPIC BeadChip microarray (850 K) and the Illumina HiScan System (Illumina). This approach allows for the quantitative detection of methylation levels at over 850,000 CpG sites across the genome, including more than 90% of the sites covered by the Illumina HumanMethylation450 BeadChip (Illumina) and over 300,000 methylation sites in enhancer regions identified by the ENCODE and FANTOM5 projects [47,73].

### 4.7. Array Data Preprocessing

The EPIC array methylation data were fully preprocessed using the *minfi* package (v.1.32.0) [74] in the R software environment (v.4.0.2). After importing the IDAT files, methylation data from sex chromosome probes were analyzed to verify the self-reported sex of the participants. Additionally, SNP/ethnicity probes from the *sesame* package (v.1.4.0) [75] were used to detect potential unwanted sources of variation. Samples that failed to meet the specified quality control criteria for intensity signals in both the methylated and unmethylated channels were excluded.

After completing the quality control steps, background noise signal was removed from the intensity values using the ssNoob method [76] in *minfi*. The extracted β values were then normalized using the β mixture quantile normalization (BMIQ) approach [77] implemented in ChAMP (v.2.16.2) [78]. Furthermore, to avoid spurious methylation signals, probes were filtered based on the following criteria: (a) having a detection *p*-value > 0.01 in any sample; (b) being cross-reactive or multi-mapping probes [72,79]; (c) being located on sex chromosomes; and (d) containing SNPs with a minor allele frequency (MAF) ≥ 0.01 at their CpG or single base extension (SBE) sites (dbSNP v.147). Finally, experiment-specific conflicting probes (*n* = 424) were identified using the clustered distribution approach implemented in the *gaphunter* function [80] of the *minfi* package (threshold = 0.20, outCutoff = 5/63) and were removed from subsequent analysis. The final number of probes that passed all filters for differential methylation analyses was 747,200 (Supplementary Materials: Figure S5).

### 4.8. cfDNA Cell-Type Deconvolution

The Houseman algorithm [81] implemented in the *ENmix* package (v.1.28.2) [82] and the FlowSorted.Blood. EPIC reference dataset [83] were used to estimate blood cell type composition from DNA methylation data. Moreover, the deconvolution algorithm and the reference atlas of Moss et al. [72] were applied to our methylome array data using Python software (v. 3.8.13) to reveal the tissular and cellular origins of cfDNA.

### 4.9. Surrogate Variable Analysis

Surrogate variable analysis was performed using the *sva* package (v.3.36.0) [84] in order to identify the main sources of variation in the high-dimensional data of EPIC array methylation. The surrogate variables identified by the Be method were then correlated with the clinicopathological features of the study participants.

### 4.10. Probe-Level Differential Methylation Analyses

Differentially methylated positions (DMPs) between patients with AD and controls were identified through the application of linear regression models, defined in the *limma* package (v.3.44.3) [85]. M-values were selected as the dependent variable in the models, since this logit transformation of methylation β-values achieves greater homoscedasticity for statistical inference [86]. Based on the results of the surrogate variable analysis, all models included the following fixed covariates: sex, age, percentage of non-sized cfDNA, batch effects (array position), and cell-type composition obtained from deconvolution analyses. Finally, empirical Bayes moderated *t* tests allowed us to perform contrasts to define DMPs, with *p*-values adjusted for multiple comparisons using the Benjamini–Hochberg method (FDR < 0.05). Differential enrichment of DMPs in relation to their genomic distribution was assessed using hypergeometric tests.

### 4.11. Region-Level Differential Methylation Analyses

To detect differentially methylated regions (DMRs), the *limma p*-values were fed in the comb p function [87] of the *ENmix* package (v.1.28.2) [82] using the default parameters. This method allowed the detection of spatially related CpG sites with statistical significance. The initial regions were first selected under an FDR < 0.05, and subsequently, the final DMRs were defined applying a Sidak corrected *p*-value threshold of < 0.05.

### 4.12. Probe Annotation

The *IlluminaHumanMethylationEPICanno.ilm10b4.hg19* package (v.0.6.0) was used to assign each probe to its corresponding location within CpG islands (CGIs) and genes. For the annotation of regions, the probes belonging to each region were first individually annotated, as described above. A single annotation was then assigned to each region according to the following criteria: 

(1) for CGI status, “Island” > “N_Shore” > “S_Shore” > “N_Shelf” > “S_Shelf” > “OpenSea”; and (2) for gene locations, “TSS1500” > “TSS200” > “5′UTR” > “1stExon” > “Body” > “3′UTR” > “Intergenic”.

### 4.13. Functional In Silico Analysis of DMPs

We employed ingenuity pathway analysis (IPA) software (v.23.0) from Ingenuity Systems^®^ (QIAGEN, Redwood City, CA, USA) to further explore the biological significance of AD-related DMPs by means of causal analytics algorithms [33]. Using this tool, we were able to identify the biological functions and diseases most significantly related to the differentially methylated genes in our dataset. The *p*-value was calculated using Fisher’s exact test. Additionally, the upstream regulator analysis was utilized to identify upstream molecules that potentially regulate the differentially methylated genes within our dataset and to construct gene networks. Simultaneously, we conducted a systematic manual annotation using PubMed to determine whether the differentially methylated genes identified in patients with AD were particularly enriched in brain functions, as previously described [16].

### 4.14. Orthogonal Validation

Furthermore, pyrosequencing and bisulfite cloning sequencing techniques were performed to validate the methylation results obtained from the microarray analysis. Briefly, the EpiTect Bisulfite Kit (QIAGEN, Redwood City, CA, USA) was used to convert 200 ng of extracted cfDNA from each sample with sodium bisulfite according to the manufacturer’s instructions.

For pyrosequencing, primers were designed with PyroMark Assay Design version 2.0.1.15 (QIAGEN, Redwood City, CA, USA), and PCR amplifications were carried out on a VeritiTM Thermal Cycler (Applied Biosystems, Foster City, CA, USA). Next, the biotinylated PCR product was captured with streptavidin-coated Sepharose beads and the sequencing primer was annealed to cfDNA strands. Pyrosequencing was carried out using PyroMark Gold Q96 reagents (QIAGEN) on a PyroMark^TM^ Q96 ID System (QIAGEN). For each CpG studied within the amplicon (CpG1-CpG4), methylation levels were expressed as the percentage of methylated cytosines relative to the total cytosines. The EpiTect PCR Control DNA Set (QIAGEN) was used as fully methylated and unmethylated DNA controls for the pyrosequencing assay.

For bisulfite cloning sequencing, the MethPrimer tool was used to design primer pair sequences [88]. PCR products were cloned using the TopoTA Cloning System (Invitrogen, Carlsbad, CA, USA), and at least 12 independent clones were sequenced by Sanger sequencing for each individual and region studied. Methylation data and graphs were obtained using QUMA software (v1.1.13) [89]. The primers used for both pyrosequencing and bisulfite cloning sequencing are provided in Supplementary Materials: Table S7.

## 5. Conclusions

In summary, our study demonstrates that cfDNA is present in the plasma of patients with AD and can be readily isolated during their lifetime. We identified 102 differentially methylated positions (DMPs) associated with AD, with up to 80% showing correlations with cognitive performance and up to 35% with established AD biomarkers. Functional analyses linked 30 DMPs to neurological processes, pinpointing critical regulators such as *SPTBN4* and the *APOE* gene. Additionally, *APOE* ε4-stratified analysis revealed six differentially methylated regions (DMRs) associated with genes previously implicated in epigenetic alterations in AD, including *HKR1*, *ZNF154*, *HOXA5*, *TRIM40*, *ATG16L2*, and *ADAMST2*. These findings provide valuable insights into the epigenetic landscape of AD and underscore the potential of cfDNA as a biomarker for advancing our understanding of the disease. The availability of blood sampling makes the analysis of epigenetic alterations in cfDNA a promising source of biomarkers in AD to be used in the practice of personalized medicine, with potential applications in identifying biomarkers, guiding targeted therapies, assessing disease risk, and linking environmental and genetic factors to optimize individualized healthcare for patients with AD. Some candidate epigenetic biomarkers seem to be related to the *APOE* genotype. Exploring the potential of liquid biopsy can enhance our understanding of this complex disorder. Moreover, this study highlights the critical importance of preanalytical factors, bioinformatics workflows, and thresholds when analyzing the cfDNA from an epigenome-wide perspective.

## Figures and Tables

**Table 1:** Blood sample set analyzed by Illumina Infinium MethylationEPIC BeadChip. The table shows the phenotypical features of the subjects included in this study. cfDNA—cell-free DNA; GDS—Global Deterioration Scale; MMSE—Mini-Mental State Examination.

Phenotypical Features	Controls (*n* = 35)	Patients with AD (*n* = 35)	*p*-Value
Median (IQR)			
Age (years)	77 (72–80)	79 (76–83)	0.213
MMSE	30 (29–30)	22 (19–26)	0.000
GDS	1 (1–1)	4 (4–4)	0.000
cfDNA amount (ng)	96 (47–212)	81 (34–241)	0.445
N (%)			
Gender			0.811
Female	17 (49)	18 (51)	
Male	18 (51)	17 (49)	
*APOE* genotype			0.001
ε4 non-carriers	31 (89)	15 (43)	
ε4 carriers	3 (9)	20 (57)	
pTau181 (pg/mL)	1.5 (1.2–1.8)	3.0 (2.1–3.9)	0.000

**Table 2:** Differentially methylated positions (DMPs) in cfDNA from patients with AD with respect to controls. The table shows 102 DMPs with difference > 0.100, prioritized by beta difference criteria. Each DMP (CpG site) was annotated by UCSC hg19 build. ID—identification.

DMP	Genomic Coordinates		Gene ID	Relation to CpG Context	Relation to Gene Structure	*p*-Value	β-Difference
cg26023019	chr21	31311859	*GRIK1*	Island	1stExon	0.013	0.155
cg19665696	chr7	949154	*ADAP1*	Island	Body	0.021	0.151
cg25069157	chr6	44102572	*TMEM63B*	OpenSea	Body	0.040	0.148
cg13578160	chr7	72813978		OpenSea		0.007	0.139
cg11955641	chr5	151304999	*GLRA1*	S_Shore	TSS1500	0.001	0.139
cg09465533	chr3	32327675	*CMTM8*	OpenSea	Body	0.001	0.137
cg20601028	chr20	22738632		OpenSea		0.006	0.133
cg24245216	chr19	7004657		OpenSea		0.044	0.130
cg23506049	chr12	103228185		OpenSea		0.039	0.129
cg21550804	chr8	74282865		OpenSea		0.035	0.125
cg22597210	chr19	10172841	*C3P1*	Island	Body	0.008	0.125
cg17857094	chr6	30907280	*DPCR1*	OpenSea	TSS1500	0.029	0.119
cg00055434	chr1	2415344	*PLCH2*	Island	Body	0.006	0.117
cg27416647	chr15	96630572		OpenSea		0.027	0.117
cg11646124	chr1	182140416		OpenSea		0.008	0.113
cg22238209	chr19	35800743	*MAG*	Island	Body	0.017	0.113
cg07983614	chr16	84587903		OpenSea		0.024	0.111
cg21764456	chr16	10777077	*TEKT5*	OpenSea	Body	0.037	0.110
cg07812827	chr8	74282708		OpenSea		0.010	0.110
cg06572225	chr11	7748353		OpenSea		0.021	0.110
cg03463818	chr8	94766468	*TMEM67*	N_Shore	TSS1500	0.034	0.108
cg26802564	chr1	30446406		OpenSea		0.006	0.107
cg18056749	chr20	55836268	*BMP7*	N_Shelf	Body	0.043	0.106
cg27454064	chr12	64215611		Island		0.017	0.105
cg24699005	chr19	1192342		N_Shelf		0.022	0.104
cg26861034	chr22	26908874	*TFIP11*	S_Shore	TSS1500	0.002	0.104
cg10411590	chr13	21900810		S_Shore		0.032	0.102
cg00796424	chr12	54365966	*HOXC11*	N_Shore	TSS1500	0.004	0.101
cg12906062	chr13	105462162		OpenSea		0.042	−0.100
cg00242341	chr11	72447419	*ARAP1*	OpenSea	5′UTR	0.039	−0.100
cg18955367	chr19	49002338	*LMTK3*	Island	Body	0.030	−0.100
cg26003334	chr7	100661866	*LOC102724094*	OpenSea	TSS1500	0.004	−0.101
cg16419584	chr10	129947858		Island		0.027	−0.101
cg13063165	chr15	79093076	*ADAMTS7*	S_Shore	Body	0.017	−0.101
cg01495416	chr8	59085270		OpenSea		0.027	−0.101
cg02258724	chr19	53832577		Island		0.020	−0.101
cg04772328	chr2	170549930	*C2orf77*	N_Shore	Body	0.002	−0.101
cg16045681	chr1	31575570		OpenSea		0.002	−0.102
cg21210642	chr9	100881995	*TRIM14*	S_Shore	TSS1500	0.008	−0.102
cg24463437	chr1	28396758	*EYA3*	OpenSea	Body	0.001	−0.102
cg20212912	chr4	147557774		N_Shore		0.007	−0.102
cg06311780	chr18	6633544		OpenSea		0.012	−0.103
cg21010821	chr11	111782679	*HSPB2*	OpenSea	TSS1500	0.004	−0.103
cg14891200	chr2	220197664	*RESP18*	S_Shore	Body	0.001	−0.103
cg14520947	chr1	225942842		OpenSea		0.003	−0.103
cg15086439	chr1	236563070	*EDARADD*	S_Shelf	Body	0.040	−0.103
cg04855678	chr3	195946921	*OSTalpha*	OpenSea	Body	0.010	−0.104
cg19146301	chr1	235100790	*LOC101927851*	OpenSea	TSS1500	0.004	−0.104
cg13443570	chr8	99098126	*ERICH5*	OpenSea	Body	0.042	−0.105
cg02784823	chr19	49000897	*LMTK3*	Island	Body	0.041	−0.105
cg06398054	chr1	53092881		OpenSea		0.019	−0.106
cg20062681	chr11	94988642		OpenSea		0.001	−0.106
cg09484559	chr17	12692246	*RICH2*	N_Shore	TSS1500	0.018	−0.106
cg08986575	chr5	173235445		OpenSea		0.049	−0.106
cg27159720	chr9	7971612		OpenSea		0.002	−0.106
cg07870920	chr4	121569769		OpenSea		0.049	−0.106
cg02938172	chr17	185152	*RPH3AL*	Island	5′UTR	0.021	−0.106
cg27087112	chr2	114737475	*LOC100499194*	Island	Body	0.000	−0.107
cg03174228	chr9	124658583	*TTLL11*	N_Shore	Body	0.010	−0.107
cg24984452	chr1	95261186	*LINC01057*	OpenSea	Body	0.035	−0.107
cg17566325	chr12	133022423		N_Shore		0.006	−0.107
cg03465894	chr11	106342311		OpenSea		0.022	−0.108
cg15410835	chr8	143125637		OpenSea		0.001	−0.108
cg24760557	chr10	31986724		OpenSea		0.016	−0.108
cg18625538	chr6	87832609		Island		0.045	−0.109
cg21933626	chr3	123026636	*ADCY5*	OpenSea	Body	0.004	−0.109
cg05800368	chr9	124658957	*TTLL11*	Island	Body	0.036	−0.109
cg02774630	chr2	154727554	*GALNT13*	N_Shore	TSS1500	0.002	−0.109
cg06878111	chr10	9999498		OpenSea		0.003	−0.110
cg23213894	chr11	7691961	*CYB5R2*	N_Shelf	Body	0.010	−0.111
cg06957053	chr7	137533035	*DGKI*	S_Shore	TSS1500	0.024	−0.111
cg10531073	chr22	38485757	*BAIAP2L2*	S_Shore	Body	0.005	−0.112
cg24104237	chr3	45649408	*LIMD1*	OpenSea	Body	0.010	−0.112
cg10289324	chr18	60710970		OpenSea		0.007	−0.113
cg15243027	chr2	32784469	*BIRC6-AS2*	OpenSea	Body	0.028	−0.113
cg10092377	chr1	200880981	*C1orf106*	Island	Body	0.001	−0.117
cg01583753	chr2	39470725		N_Shore		0.018	−0.117
cg16127514	chr10	29273678		OpenSea		0.042	−0.117
cg26496930	chr14	70186565		OpenSea		0.041	−0.117
cg10305928	chr10	62426219	*ANK3*	OpenSea	Body	0.043	−0.117
cg14310109	chr6	157297510	*ARID1B*	OpenSea	Body	0.005	−0.119
cg16520701	chr8	34606956		OpenSea		0.010	−0.120
cg16210088	chr22	31318349	*C22orf27*	Island	Body	0.009	−0.122
cg24448113	chr5	140475611	*PCDHB2*	Island	1stExon	0.013	−0.124
cg06862049	chr19	49001890	*LMTK3*	Island	Body	0.023	−0.125
cg12172631	chr19	54584915	*TARM1*	OpenSea	TSS1500	0.021	−0.126
cg20548231	chr22	31318373	*C22orf27*	Island	Body	0.001	−0.127
cg09544050	chr8	143580965	*BAI1*	Island	Body	0.003	−0.128
cg18815398	chr20	61506981		N_Shore		0.000	−0.128
cg26651782	chr19	51505779	*KLK9*	N_Shore	3′UTR	0.005	−0.128
cg24061197	chr2	220108496	*GLB1L*	S_Shore	5′UTR	0.043	−0.128
cg06260707	chr1	42945689		OpenSea		0.022	−0.128
cg15059639	chr2	171220061	*MYO3B*	OpenSea	Body	0.022	−0.128
cg24658778	chr6	152897280	*SYNE1*	OpenSea	Body	0.013	−0.130
cg20920357	chr4	116877727		OpenSea		0.002	−0.133
cg02256650	chr22	31317287	*MORC2-AS1*	N_Shore	TSS1500	0.013	−0.133
cg26140120	chr8	124219575	*FAM83A*	Island	Body	0.014	−0.135
cg06452258	chr2	60597809		OpenSea		0.049	−0.136
cg08431893	chr21	44864600		Island		0.004	−0.139
cg16467921	chr8	128801108		OpenSea		0.004	−0.139
cg04248279	chr17	184833	*RPH3AL*	N_Shore	5′UTR	0.007	−0.142
cg24135491	chr17	4487099	*SMTNL2*	N_Shore	TSS200	0.015	−0.147

**Table 3:** Correlation between DNA methylation of 10 top differential methylated positions (DMPs) ranked by highest positive beta difference (delta mean) criteria and clinical parameters. ID—identification; MMSE—Mini-Mental State Examination; GDS—Global Deterioration Scale. ^+^ *p*-value < 0.1; * *p*-value < 0.05; ** *p*-value < 0.01.

No	DMP	β-Difference	Gene ID	MMSE	GDS	Aβ42	Aβ40	Ratio Aβ42/Aβ40	pTau	t-Tau	Plasma pTau181
1	cg26023019	0.155	*GRIK1*	−0.155	0.224	−0.044	−0.032	−0.102	0.290	0.096	0.148
2	cg19665696	0.151	*ADAP1*	−0.204	0.328 **	−0.180	0.093	−0.549 *	0.075	−0.162	0.329 *
3	cg25069157	0.148	*TMEM63B*	−0.242	−0.204	0.156	0.071	0.039	−0.164	0.048	0.378 **
4	cg13578160	0.139		−0.336 *	0.338 **	−0.050	−0.003	0.086	−0.050	0.033	0.117
5	cg11955641	0.139	*GLRA1*	−0.278 *	0.452 **	0.275	0.444 ^+^	−0.081	0.403 ^+^	0.268	0.327 *
6	cg09465533	0.137	*CMTM8*	−0.304 *	0.413 **	−0.331	−0.233	−0.149	0.107	−0.117	0.141
7	cg20601028	0.133		−0.317 *	0.352 **	−0.036	−0.024	−0.116	0.221	−0.053	0.330 *
8	cg24245216	0.130		−0.349 **	0.273 *	−0.353	−0.659 **	0.332	−0.427 ^+^	−0.513 *	0.229 ^+^
9	cg23506049	0.129		−0.502 **	0.373 **	0.177	−0.029	0.159	−0.117	−0.313	0.199
10	cg21550804	0.125		−0.167	0.137	0.174	0.093	0.302	0.302	0.347	0.283 *

**Table 4:** Correlation between DNA methylation of 10 top differential methylated positions (DMPs) ranked by highest negative beta difference (delta) criteria and clinical parameters. ID—identification; MMSE—Mini-Mental State Examination; GDS—Global Deterioration Scale. ^+^ *p*-value < 0.1; * *p*-value < 0.05; ** *p*-value < 0.01.

No	DMP	β-Difference	Gene ID	MMSE	GDS	Aβ42	Aβ40	Ratio Aβ42/Aβ40	pTau	t-Tau	Plasma pTau181
1	cg24135491	−0.147	*SMTNL2*	0.349 **	−0.463 **	−0.302	−0.205	−0.092	−0.155	−0.308	−0.357 **
2	cg04248279	−0.142	*RPH3AL*	0.202	−0.233	0.188	−0.008	0.176	−0.326	−0.317	−0.361 **
3	cg16467921	−0.139		0.258	−0.346 **	−0.048	0.092	−0.448 *	0.301	0.134	−0.227 ^+^
4	cg08431893	−0.139		0.278 *	−0.338 **	−0.170	−0.198	0.041	−0.119	−0.122	−0.265 ^+^
5	cg06452258	−0.136		0.341 *	0.430 **	−0.084	0.039	−0.101	0.039	0.122	−0.231 ^+^
6	cg26140120	−0.135	*FAM83A*	0.517 **	−0.373 **	0.135	0.092	0.156	0.032	0.233	−0.217
7	cg02256650	−0.133	*MORC2-AS1*	0.299 *	−0.409 **	−0.092	−0.026	0.080	0.036	0.189	−0.253 ^+^
8	cg20920357	−0.133		0.040	−0.312 *	0.397 ^+^	0.365	0.165	0.071	0.095	−0.194
9	cg24658778	−0.130	*SYNE1*	0.311 *	−0.296 *	−0.003	0.185	−0.096	0.253	0.098	−0.159
10	cg15059639	−0.128	*MYO3B*	0.203	−0.252 *	0.092	0.173	0.003	0.105	0.021	−0.223 ^+^

**Table 5:** Differentially methylated regions (DMRs) in cfDNA from AD *APOE* ε4 carriers and control *APOE* ε4 non-carriers. The table shows 17 DMRs after adjusting for Sidak correction, prioritized by beta difference criteria. Each region was annotated by UCSC hg19 build.

Genomic Location			FDR	Sidak	CpGs in DMR	Gene ID	Relation to CpG Context	Relation to Gene Structure	β-Difference
chr8	69243284	69243293	2.41099 × 10^−6^	0.021	cg06125462; cg03357798; cg19469068	*C8orf34; C8orf34-AS1*	Island	1stExon; Intergenic	0.038
chr11	89867808	89868104	6.55924 × 10^−6^	0.004	cg14304817; cg09584827; cg12403137; cg05236757; cg05500015; cg21500966	*NAALAD2*	OpenSea	5′UTR; TSS200; Body; 1stExon	0.013
chr19	55972854	55973234	2.06981× 10^−5^	0.026	cg12195369; cg02394686; cg23051792; cg18297529; cg19384825;cg13475732	*ISOC2*	Island	5′UTR; TSS200	−0.004
chr2	168366268	168366363	7.3012 × 10^−6^	0.021	cg19288676; cg18683711		OpenSea	Intergenic	−0.016
chr19	1854548	1854819	6.55924 × 10^−6^	0.005	cg08287334; cg22672431; cg08525314; cg01373896	*KLF16*	Island	Body	−0.026
chr6	116381903	116382179	3.57858 × 10^−6^	0.002	cg15226275; cg05304507; cg26893134; cg26557270; cg18764771	*FRK*	OpenSea	TSS200; 1stExon; TSS1500	−0.027
chr5	1316037	1316264	8.74148 × 10^−6^	0.017	cg11624060; cg26209169		OpenSea	Intergenic	−0.033
chr1	155007165	155007254	8.18998 × 10^−6^	0.038	cg08472142; cg21596858	*DCST1;DCST2*	OpenSea	Body	−0.038
chr5	178692690	178692806	8.18998 × 10^−6^	0.028	cg06495631; cg01231141; cg10213542	*ADAMTS2*	OpenSea	Body	−0.040
chr6	30103458	30103699	7.53684 × 10^−6^	0.010	cg12758147; cg12612406; cg13044052	*TRIM40*	OpenSea	TSS1500	−0.044
chr20	31366408	31366486	8.18998 × 10^−6^	0.040	cg00300969;cg09135144; cg24403338; cg17475857	*DNMT3B*	OpenSea	5′UTR; TSS1500	−0.046
chr11	72533201	72533487	8.18998 × 10^−6^	0.011	cg13771313; cg24878173; cg04006327	*ATG16L2*	Island	Body	−0.057
chr19	7983876	7984171	1.60512 × 10^−7^	0.000	cg26284544; cg10073052; cg00654322; cg03685315; cg03840143	*SNAPC2*	Island; S_Shore	TSS1500	−0.058
chr10	123070326	123070392	7.53684 × 10^−6^	0.036	cg16273546;cg21380925		OpenSea	Intergenic	−0.064
chr6	30419490	30419576	1.45527 × 10^−15^	0.000	cg26570901; cg26715559; cg12078775; cg27572120;cg11491998		Island	Intergenic	−0.074
chr5	1867977	1868261	8.18998 × 10^−6^	0.012	cg15595755; cg04156016; cg14773178		OpenSea	Intergenic	−0.084
chr2	220108093	220108496	2.16849 × 10^−6^	0.000	cg20314884; cg04945312; cg10602248; cg02258512; cg24061197; cg09715285	*GLB1L*	Island; S_Shore	Body; 5′UTR; 1stExon	−0.095

**Table 6:** Differentially methylated regions (DMRs) in cfDNA from AD APOE ε4 carriers and control APOE ε4 non-carriers. The table shows 4 DMRs after adjusting for Sidak correction, prioritized by beta difference criteria. Each region was annotated by UCSC hg19 build.

Genomic Location			FDR	Sidak	CpGs in DMR	Gene ID	Relation to CpG Context	Relation to Gene Structure	β-Difference
chr13	21900391	21900591	2.318 × 10^−6^	4.081 × 10^−3^	cg13903179; cg19500098; cg00035636; cg04632378		Island	Intergenic	0.100
chr17	45949676	45949878	2.318 × 10^−6^	4.887 × 10^−3^	cg01135546; cg09065876; cg16913064; cg23008871; cg05123976		Island	Intergenic	0.037
chr19	37825210	37825679	1.743 × 10^−8^	7.935 × 10^−6^	cg26734888; cg23756236; cg12948621; cg12024906; cg08565796; cg24834889; cg14166009; cg10237978; cg05280698; cg13687570	*HKR1*	Island	TSS1500; TSS200; 1stExon	0.021
chr3	133748504	133748812	4.661 × 10^−10^	1.615 × 10^−7^	cg19591206; cg12118082; cg26556923; cg02496728	*SLCO2A1*	Island	1stExon; Body	0.010

**Table 7:** Differentially methylated genes reported in previous Alzheimer’s disease (AD) methylome studies. The table shows the genes that have been previously found associated with AD in methylome studies performed on different sample sources and techniques. ID—identification.

Gene ID	Sample Source	Technique	AD Methylome Study
*HKR1*	Hippocampus	Infinium HumanMethylation450 BeadChip	PMID: 31217032
	Prefrontal cortex	Infinium MethylationEPIC BeadChip	PMID: 33257653
	Blood	Infinium HumanMethylation450 BeadChip	PMID: 29394898
*ZNF154*	Blood	Infinium HumanMethylation450 BeadChip	PMID: 31775875
*HOXA5*	Superior temporal gyrus and inferior frontal gyrus	Infinium MethylationEPIC BeadChip	PMID: 33069246
	Prefrontal cortex	Infinium HumanMethylation450 BeadChip	PMID: 33902726
	Superior temporal gyrus	Infinium HumanMethylation450 BeadChip	PMID: 29550519
*TRIM40*	Superior temporal gyrus	Infinium HumanMethylation450 BeadChip	PMID: 26803900
	Prefrontal cortex	Infinium MethylationEPIC BeadChip	PMID: 33257653
*ATG16L2*	Hippocampus	Infinium HumanMethylation450 BeadChip	PMID: 31217032
	Dorsolateral prefrontal cortex	Infinium HumanMethylation450 BeadChip	PMID: 25129075
	Prefrontal cortex	Infinium HumanMethylation450 BeadChip	PMID: 33902726
	Prefrontal cortex	Infinium MethylationEPIC BeadChip	PMID: 33257653
*ADAMST2*	Prefrontal cortex	Infinium HumanMethylation450 BeadChip	PMID: 35982059

## Data Availability

All data generated and/or analyzed during this study are either included in this article or are available from the corresponding author on reasonable request.

## Supplementary Materials

The following supporting information can be downloaded at: https://www.mdpi.com/article/10.3390/ijms26073419/s1.

## Abbreviations

The following abbreviations are used in this manuscript:

AD	Alzheimer’s disease
APOE	Apolipoprotein E
cfDNA	Cell-free DNA
CpG	Cytosine guanine dinucleotide
CSF	Cerebrospinal fluid
DMP	Differential methylated position
DMR	Differential methylated region
GDS	Global Deterioration Scale
LOAD	Late-onset Alzheimer’s disease
MMSE	Mini-Mental State Examination

## References

[1] (2024). 2024 Alzheimer’s disease facts and figures. Alzheimers Dement..

[2] Alzheimers Disease International (2018). World Alzheimer Report 2018. https://www.alz.co.uk/research/WorldAlzheimerReport2018.pdf.

[3] Alzheimers Disease International (2021). World Alzheimer Report 2021. https://www.alzint.org/u/World-Alzheimer-Report-2021.pdf.

[4] Lambert J.C., Ibrahim-Verbaas C.A., Harold D., Naj A.C., Sims R., Bellenguez C., DeStafano A.L., Bis J.C., Beecham G.W., Grenier-Boley B. (2013). Meta-analysis of 74,046 individuals identifies 11 new susceptibility loci for Alzheimer’s disease. Nat. Genet..

[5] Sanchez-Mut J.V., Gräff J. (2015). Epigenetic Alterations in Alzheimer’s Disease. Front. Behav. Neurosci..

[6] Zhang L., Silva T.C., Young J.I., Gomez L., Schmidt M.A., Hamilton-Nelson K.L., Kunkle B.W., Chen X., Martin E.R., Wang L. (2020). Epigenome-wide meta-analysis of DNA methylation differences in prefrontal cortex implicates the immune processes in Alzheimer’s disease. Nat. Commun..

[7] Zhang L., Young J.I., Gomez L., Silva T.C., Schmidt M.A., Cai J., Chen X., Martin E.R., Wang L. (2021). Sex-specific DNA methylation differences in Alzheimer’s disease pathology. Acta Neuropathol. Commun..

[8] De Jager P.L., Srivastava G., Lunnon K., Burgess J., Schalkwyk L.C., Yu L., Eaton M.L., Keenan B.T., Ernst J., McCabe C. (2014). Alzheimer’s disease: Early alterations in brain DNA methylation at ANK1, BIN1, RHBDF2 and other loci. Nat. Neurosci..

[9] Lunnon K., Smith R., Hannon E., De Jager P.L., Srivastava G., Volta M., Troakes C., Al-Sarraj S., Burrage J., Macdonald R. (2014). Methylomic profiling implicates cortical deregulation of ANK1 in Alzheimer’s disease. Nat. Neurosci..

[10] Gasparoni G., Bultmann S., Lutsik P., Kraus T.F.J., Sordon S., Vlcek J., Dietinger V., Steinmaurer M., Haider M., Mulholland C.B. (2018). DNA methylation analysis on purified neurons and glia dissects age and Alzheimer’s disease-specific changes in the human cortex. Epigenetics Chromatin.

[11] Smith R.G., Pishva E., Shireby G., Smith A.R., Roubroeks J.A.Y., Hannon E., Wheildon G., Mastroeni D., Gasparoni G., Riemenschneider M. (2021). A meta-analysis of epigenome-wide association studies in Alzheimer’s disease highlights novel differentially methylated loci across cortex. Nat. Commun..

[12] Sanchez-Mut J.V., Heyn H., Vidal E., Delgado-Morales R., Moran S., Sayols S., Sandoval J., Ferrer I., Esteller M., Gräff J. (2017). Whole genome grey and white matter DNA methylation profiles in dorsolateral prefrontal cortex. Synapse.

[13] Smith R.G., Hannon E., De Jager P.L., Chibnik L., Lott S.J., Condliffe D., Smith A.R., Haroutunian V., Troakes C., Al-Sarraj S. (2018). Elevated DNA methylation across a 48-kb region spanning the HOXA gene cluster is associated with Alzheimer’s disease neuropathology. Alzheimers Dement..

[14] Semick S.A., Bharadwaj R.A., Collado-Torres L., Tao R., Shin J.H., Deep-Soboslay A., Weiss J.R., Weinberger D.R., Hyde T.M., Kleinman J.E. (2019). Integrated DNA methylation and gene expression profiling across multiple brain regions implicate novel genes in Alzheimer’s disease. Acta Neuropathol..

[15] Rao J.S., Keleshian V.L., Klein S., Rapoport S.I. (2012). Epigenetic modifications in frontal cortex from Alzheimer’s disease and bipolar disorder patients. Transl. Psychiatry.

[16] Altuna M., Urdanoz-Casado A., Sanchez-Ruiz de Gordoa J., Zelaya M.V., Labarga A., Lepesant J.M.J., Roldan M., Blanco-Luquin I., Perdones A., Larumbe R. (2019). DNA methylation signature of human hippocampus in Alzheimer’s disease is linked to neurogenesis. Clin. Epigenetics.

[17] Li Q.S., Sun Y., Wang T. (2020). Epigenome-wide association study of Alzheimer’s disease replicates 22 differentially methylated positions and 30 differentially methylated regions. Clin. Epigenetics.

[18] Watson C.T., Roussos P., Garg P., Ho D.J., Azam N., Katsel P.L., Haroutunian V., Sharp A.J. (2016). Genome-wide DNA methylation profiling in the superior temporal gyrus reveals epigenetic signatures associated with Alzheimer’s disease. Genome Med..

[19] Perez R.F., Alba-Linares J.J., Tejedor J.R., Fernandez A.F., Calero M., Roman-Dominguez A., Borras C., Vina J., Avila J., Medina M. (2022). Blood DNA Methylation Patterns in Older Adults With Evolving Dementia. J. Gerontol. A Biol. Sci. Med. Sci..

[20] Lardenoije R., Roubroeks J.A.Y., Pishva E., Leber M., Wagner H., Iatrou A., Smith A.R., Smith R.G., Eijssen L.M.T., Kleineidam L. (2019). Alzheimer’s disease-associated (hydroxy)methylomic changes in the brain and blood. Clin. Epigenetics.

[21] Konki M., Malonzo M., Karlsson I.K., Lindgren N., Ghimire B., Smolander J., Scheinin N.M., Ollikainen M., Laiho A., Elo L.L. (2019). Peripheral blood DNA methylation differences in twin pairs discordant for Alzheimer’s disease. Clin. Epigenetics.

[22] Chang L., Wang Y., Ji H., Dai D., Xu X., Jiang D., Hong Q., Ye H., Zhang X., Zhou X. (2014). Elevation of peripheral BDNF promoter methylation links to the risk of Alzheimer’s disease. PLoS ONE.

[23] Macías M., Alegre E., Díaz-Lagares A., Patiño A., Pérez-Gracia J.L., Sanmamed M., López-López R., Varo N., González A. (2018). Liquid Biopsy: From Basic Research to Clinical Practice. Adv. Clin. Chem..

[24] Sun K., Jiang P., Chan K.C., Wong J., Cheng Y.K., Liang R.H., Chan W.K., Ma E.S., Chan S.L., Cheng S.H. (2015). Plasma DNA tissue mapping by genome-wide methylation sequencing for noninvasive prenatal, cancer, and transplantation assessments. Proc. Natl. Acad. Sci. USA.

[25] Lehmann-Werman R., Neiman D., Zemmour H., Moss J., Magenheim J., Vaknin-Dembinsky A., Rubertsson S., Nellgård B., Blennow K., Zetterberg H. (2016). Identification of tissue-specific cell death using methylation patterns of circulating DNA. Proc. Natl. Acad. Sci. USA.

[26] Gaitsch H., Franklin R.J.M., Reich D.S. (2022). Cell-free DNA-based liquid biopsies in neurology. Brain.

[27] Southwood D., Singh S., Chatterton Z. (2022). Brain-derived cell-free DNA. Neural Regen. Res..

[28] Khemka S., Sehar U., Manna P.R., Kshirsagar S., Reddy P.H. (2024). Cell-Free DNA As Peripheral Biomarker of Alzheimer’s Disease. Aging Dis..

[29] Pollard C., Aston K., Emery B.R., Hill J., Jenkins T. (2023). Detection of neuron-derived cfDNA in blood plasma: A new diagnostic approach for neurodegenerative conditions. Front. Neurol..

[30] Noe C.R., Noe-Letschnig M., Handschuh P., Noe C.A., Lanzenberger R. (2020). Dysfunction of the Blood-Brain Barrier-A Key Step in Neurodegeneration and Dementia. Front. Aging Neurosci..

[31] Zenaro E., Piacentino G., Constantin G. (2017). The blood-brain barrier in Alzheimer’s disease. Neurobiol. Dis..

[32] Costa-Pinheiro P., Montezuma D., Henrique R., Jerónimo C. (2015). Diagnostic and prognostic epigenetic biomarkers in cancer. Epigenomics.

[33] Krämer A., Green J., Pollard J., Tugendreich S. (2014). Causal analysis approaches in Ingenuity Pathway Analysis. Bioinformatics.

[34] Walker R.M., Vaher K., Bermingham M.L., Morris S.W., Bretherick A.D., Zeng Y., Rawlik K., Amador C., Campbell A., Haley C.S. (2021). Identification of epigenome-wide DNA methylation differences between carriers of APOE epsilon4 and APOE epsilon2 alleles. Genome Med..

[35] Darst R.P., Pardo C.E., Ai L., Brown K.D., Kladde M.P. (2010). Bisulfite sequencing of DNA. Curr. Protoc. Mol. Biol..

[36] Gallardo-Gomez M., Moran S., Paez de la Cadena M., Martinez-Zorzano V.S., Rodriguez-Berrocal F.J., Rodriguez-Girondo M., Esteller M., Cubiella J., Bujanda L., Castells A. (2018). A new approach to epigenome-wide discovery of non-invasive methylation biomarkers for colorectal cancer screening in circulating cell-free DNA using pooled samples. Clin. Epigenetics.

[37] Siravegna G., Marsoni S., Siena S., Bardelli A. (2017). Integrating liquid biopsies into the management of cancer. Nat. Rev. Clin. Oncol..

[38] Pai M.C., Kuo Y.M., Wang I.F., Chiang P.M., Tsai K.J. (2019). The Role of Methylated Circulating Nucleic Acids as a Potential Biomarker in Alzheimer’s Disease. Mol. Neurobiol..

[39] Mendioroz M., Martínez-Merino L., Blanco-Luquin I., Urdánoz A., Roldán M., Jericó I. (2018). Liquid biopsy: A new source of candidate biomarkers in amyotrophic lateral sclerosis. Ann. Clin. Transl. Neurol..

[40] Bronkhorst A.J., Aucamp J., Pretorius P.J. (2015). Cell-free DNA: Preanalytical variables. Clin. Chim. Acta.

[41] Kresse S.H., Brandt-Winge S., Pharo H., Flatin B.T.B., Jeanmougin M., Vedeld H.M., Lind G.E. (2023). Evaluation of commercial kits for isolation and bisulfite conversion of circulating cell-free tumor DNA from blood. Clin. Epigenetics.

[42] Alcaide M., Cheung M., Hillman J., Rassekh S.R., Deyell R.J., Batist G., Karsan A., Wyatt A.W., Johnson N., Scott D.W. (2020). Evaluating the quantity, quality and size distribution of cell-free DNA by multiplex droplet digital PCR. Sci. Rep..

[43] Guemri J., Pierre-Jean M., Brohard S., Oussada N., Horgues C., Bonnet E., Mauger F., Deleuze J.F. (2022). Methylated ccfDNA from plasma biomarkers of Alzheimer’s disease using targeted bisulfite sequencing. Epigenomics.

[44] Chen L., Shen Q., Xu S., Yu H., Pei S., Zhang Y., He X., Wang Q., Li D. (2022). 5-Hydroxymethylcytosine Signatures in Circulating Cell-Free DNA as Diagnostic Biomarkers for Late-Onset Alzheimer’s Disease. J. Alzheimers Dis..

[45] Bahado-Singh R.O., Radhakrishna U., Gordevicius J., Aydas B., Yilmaz A., Jafar F., Imam K., Maddens M., Challapalli K., Metpally R.P. (2022). Artificial Intelligence and Circulating Cell-Free DNA Methylation Profiling: Mechanism and Detection of Alzheimer’s Disease. Cells.

[46] Bahado-Singh R.O., Vishweswaraiah S., Turkoglu O., Graham S.F., Radhakrishna U. (2023). Alzheimer’s Precision Neurology: Epigenetics of Cytochrome P450 Genes in Circulating Cell-Free DNA for Disease Prediction and Mechanism. Int. J. Mol. Sci..

[47] Pidsley R., Zotenko E., Peters T.J., Lawrence M.G., Risbridger G.P., Molloy P., Van Djik S., Muhlhausler B., Stirzaker C., Clark S.J. (2016). Critical evaluation of the Illumina MethylationEPIC BeadChip microarray for whole-genome DNA methylation profiling. Genome Biol..

[48] Konki M., Lindgren N., Kylaniemi M., Venho R., Laajala E., Ghimire B., Lahesmaa R., Kaprio J., Rinne J.O., Lund R.J. (2020). Plasma cell-free DNA methylation marks for episodic memory impairment: A pilot twin study. Sci. Rep..

[49] Huang C.Y., Rasband M.N. (2018). Axon initial segments: Structure, function, and disease. Ann. N. Y. Acad. Sci..

[50] Grubb M.S., Burrone J. (2010). Building and maintaining the axon initial segment. Curr. Opin. Neurobiol..

[51] Sanchez-Mut J.V., Aso E., Panayotis N., Lott I., Dierssen M., Rabano A., Urdinguio R.G., Fernandez A.F., Astudillo A., Martin-Subero J.I. (2013). DNA methylation map of mouse and human brain identifies target genes in Alzheimer’s disease. Brain.

[52] Zhu D., Li C., Swanson A.M., Villalba R.M., Guo J., Zhang Z., Matheny S., Murakami T., Stephenson J.R., Daniel S. (2015). BAI1 regulates spatial learning and synaptic plasticity in the hippocampus. J. Clin. Investig..

[53] Foraker J., Millard S.P., Leong L., Thomson Z., Chen S., Keene C.D., Bekris L.M., Yu C.E. (2015). The APOE Gene is Differentially Methylated in Alzheimer’s Disease. J. Alzheimers Dis..

[54] Panitch R., Sahelijo N., Hu J., Nho K., Bennett D.A., Lunetta K.L., Au R., Stein T.D., Farrer L.A., Jun G.R. (2024). APOE genotype-specific methylation patterns are linked to Alzheimer disease pathology and estrogen response. Transl. Psychiatry.

[55] Choi K., Zhuang H., Crain B., Doré S. (2008). Expression and localization of prostaglandin transporter in Alzheimer disease brains and age-matched controls. J. Neuroimmunol..

[56] Gagliardi M., Strazzullo M., Matarazzo M.R. (2018). DNMT3B Functions: Novel Insights From Human Disease. Front. Cell Dev. Biol..

[57] Rajendran G., Shanmuganandam K., Bendre A., Muzumdar D., Goel A., Shiras A. (2011). Epigenetic regulation of DNA methyltransferases: DNMT1 and DNMT3B in gliomas. J. Neurooncol..

[58] Wang K., Liu H., Hu Q., Wang L., Liu J., Zheng Z., Zhang W., Ren J., Zhu F., Liu G.H. (2022). Epigenetic regulation of aging: Implications for interventions of aging and diseases. Signal Transduct. Target. Ther..

[59] de Bem C.M., Pezzi J.C., Borba E.M., Chaves M.L., de Andrade F.M., Fiegenbaum M., Camozzato A. (2016). The synergistic risk effect of apolipoprotein epsilon4 and DNA (cytosine-5-)-methyltransferase 3 beta (DNMT3B) haplotype for Alzheimer’s disease. Mol. Biol. Rep..

[60] Tannorella P., Stoccoro A., Tognoni G., Petrozzi L., Salluzzo M.G., Ragalmuto A., Siciliano G., Haslberger A., Bosco P., Bonuccelli U. (2015). Methylation analysis of multiple genes in blood DNA of Alzheimer’s disease and healthy individuals. Neurosci. Lett..

[61] Zeng Q., Chen X., Ning C., Zhu Q., Yao Y., Zhao Y., Luan F. (2018). Methylation of the genes ROD1, NLRC5, and HKR1 is associated with aging in Hainan centenarians. BMC Med. Genom..

[62] Berson A., Nativio R., Berger S.L., Bonini N.M. (2018). Epigenetic Regulation in Neurodegenerative Diseases. Trends Neurosci..

[63] Lardenoije R., Iatrou A., Kenis G., Kompotis K., Steinbusch H.W., Mastroeni D., Coleman P., Lemere C.A., Hof P.R., van den Hove D.L. (2015). The epigenetics of aging and neurodegeneration. Prog. Neurobiol..

[64] Olmedillas-López S., Olivera-Salazar R., García-Arranz M., García-Olmo D. (2022). Current and Emerging Applications of Droplet Digital PCR in Oncology: An Updated Review. Mol. Diagn. Ther..

[65] Nidadavolu L.S., Feger D., Wu Y., Grodstein F., Gross A.L., Bennett D.A., Walston J.D., Oh E.S., Abadir P.M. (2022). Circulating Cell-Free Genomic DNA Is Associated with an Increased Risk of Dementia and with Change in Cognitive and Physical Function. J. Alzheimers Dis..

[66] Wenz H.M., Dailey D., Johnson M.D. (2001). Development of a high-throughput capillary electrophoresis protocol for DNA fragment analysis. Methods Mol. Biol..

[67] Jack C.R., Bennett D.A., Blennow K., Carrillo M.C., Dunn B., Haeberlein S.B., Holtzman D.M., Jagust W., Jessen F., Karlawish J. (2018). NIA-AA Research Framework: Toward a biological definition of Alzheimer’s disease. Alzheimers Dement..

[68] Folstein M.F., Folstein S.E., McHugh P.R. (1975). “Mini-mental state”. A practical method for grading the cognitive state of patients for the clinician. J. Psychiatr. Res..

[69] Reisberg B., Ferris S.H., de Leon M.J., Crook T. (1982). The Global Deterioration Scale for assessment of primary degenerative dementia. Am. J. Psychiatry.

[70] Leon S.A., Shapiro B., Sklaroff D.M., Yaros M.J. (1977). Free DNA in the serum of cancer patients and the effect of therapy. Cancer Res..

[71] Stevenson M., Nunes T., Sanchez J., Thornton R., Reiczigel J., Robison-Cox J., Sebastiani P. (2013). EpiR: An R Package for the Analysis of Epidemiological Data. https://www.researchgate.net/publication/303185003_EpiR_An_R_package_for_the_analysis_of_epidemiological_data.

[72] Moss J., Magenheim J., Neiman D., Zemmour H., Loyfer N., Korach A., Samet Y., Maoz M., Druid H., Arner P. (2018). Comprehensive human cell-type methylation atlas reveals origins of circulating cell-free DNA in health and disease. Nat. Commun..

[73] Zhou W., Laird P.W., Shen H. (2017). Comprehensive characterization, annotation and innovative use of Infinium DNA methylation BeadChip probes. Nucleic Acids Res..

[74] Aryee M.J., Jaffe A.E., Corrada-Bravo H., Ladd-Acosta C., Feinberg A.P., Hansen K.D., Irizarry R.A. (2014). Minfi: A flexible and comprehensive Bioconductor package for the analysis of Infinium DNA methylation microarrays. Bioinformatics.

[75] Zhou W., Triche T.J., Laird P.W., Shen H. (2018). SeSAMe: Reducing artifactual detection of DNA methylation by Infinium BeadChips in genomic deletions. Nucleic Acids Res..

[76] Triche T.J., Weisenberger D.J., Van Den Berg D., Laird P.W., Siegmund K.D. (2013). Low-level processing of Illumina Infinium DNA Methylation BeadArrays. Nucleic Acids Res..

[77] Teschendorff A.E., Marabita F., Lechner M., Bartlett T., Tegner J., Gomez-Cabrero D., Beck S. (2013). A beta-mixture quantile normalization method for correcting probe design bias in Illumina Infinium 450 k DNA methylation data. Bioinformatics.

[78] Tian Y., Morris T.J., Webster A.P., Yang Z., Beck S., Feber A., Teschendorff A.E. (2017). ChAMP: Updated methylation analysis pipeline for Illumina BeadChips. Bioinformatics.

[79] Chen Y.A., Lemire M., Choufani S., Butcher D.T., Grafodatskaya D., Zanke B.W., Gallinger S., Hudson T.J., Weksberg R. (2013). Discovery of cross-reactive probes and polymorphic CpGs in the Illumina Infinium HumanMethylation450 microarray. Epigenetics.

[80] Andrews S.V., Ladd-Acosta C., Feinberg A.P., Hansen K.D., Fallin M.D. (2016). “Gap hunting” to characterize clustered probe signals in Illumina methylation array data. Epigenetics Chromatin.

[81] Houseman E.A., Accomando W.P., Koestler D.C., Christensen B.C., Marsit C.J., Nelson H.H., Wiencke J.K., Kelsey K.T. (2012). DNA methylation arrays as surrogate measures of cell mixture distribution. BMC Bioinform..

[82] Xu Z., Niu L., Li L., Taylor J.A. (2016). ENmix: A novel background correction method for Illumina HumanMethylation450 BeadChip. Nucleic Acids Res..

[83] Salas L.A., Koestler D.C., Butler R.A., Hansen H.M., Wiencke J.K., Kelsey K.T., Christensen B.C. (2018). An optimized library for reference-based deconvolution of whole-blood biospecimens assayed using the Illumina HumanMethylationEPIC BeadArray. Genome Biol..

[84] Leek J.T., Johnson W.E., Parker H.S., Jaffe A.E., Storey J.D. (2012). The sva package for removing batch effects and other unwanted variation in high-throughput experiments. Bioinformatics.

[85] Ritchie M.E., Phipson B., Wu D., Hu Y., Law C.W., Shi W., Smyth G.K. (2015). limma powers differential expression analyses for RNA-sequencing and microarray studies. Nucleic Acids Res..

[86] Du P., Zhang X., Huang C.-C., Jafari N., Kibbe W.A., Hou L., Lin S.M. (2010). Comparison of Beta-value and M-value methods for quantifying methylation levels by microarray analysis. BMC Bioinform..

[87] Pedersen B.S., Schwartz D.A., Yang I.V., Kechris K.J. (2012). Comb-p: Software for combining, analyzing, grouping and correcting spatially correlated P-values. Bioinformatics.

[88] Li L.C., Dahiya R. (2002). MethPrimer: Designing primers for methylation PCRs. Bioinformatics.

[89] Kumaki Y., Oda M., Okano M. (2008). QUMA: Quantification tool for methylation analysis. Nucleic Acids Res..

