# Supplementary material for: Advancing Personalized Medicine in Alzheimer’s Disease: Liquid Biopsy Epigenomics Unveil APOE ε4-Linked Methylation Signatures
Source: Int J Mol Sci. 2025 Apr 5;26(7):3419. doi: 10.3390/ijms26073419 (PMC11989983; doi:10.3390/ijms26073419)

Figure S1: Characterization of cfDNA: fragment size analysis. The diagram represents the typical pattern of cfDNA with a peak around 165 bp after performing capillary electrophoresis on a Fragment Analyzer Automated CE System with DNF-477 High Small Fragment Analysis Kit. This kit allows tracking cfDNA fragmentation pattern from 50 bp – 1,500 bp.

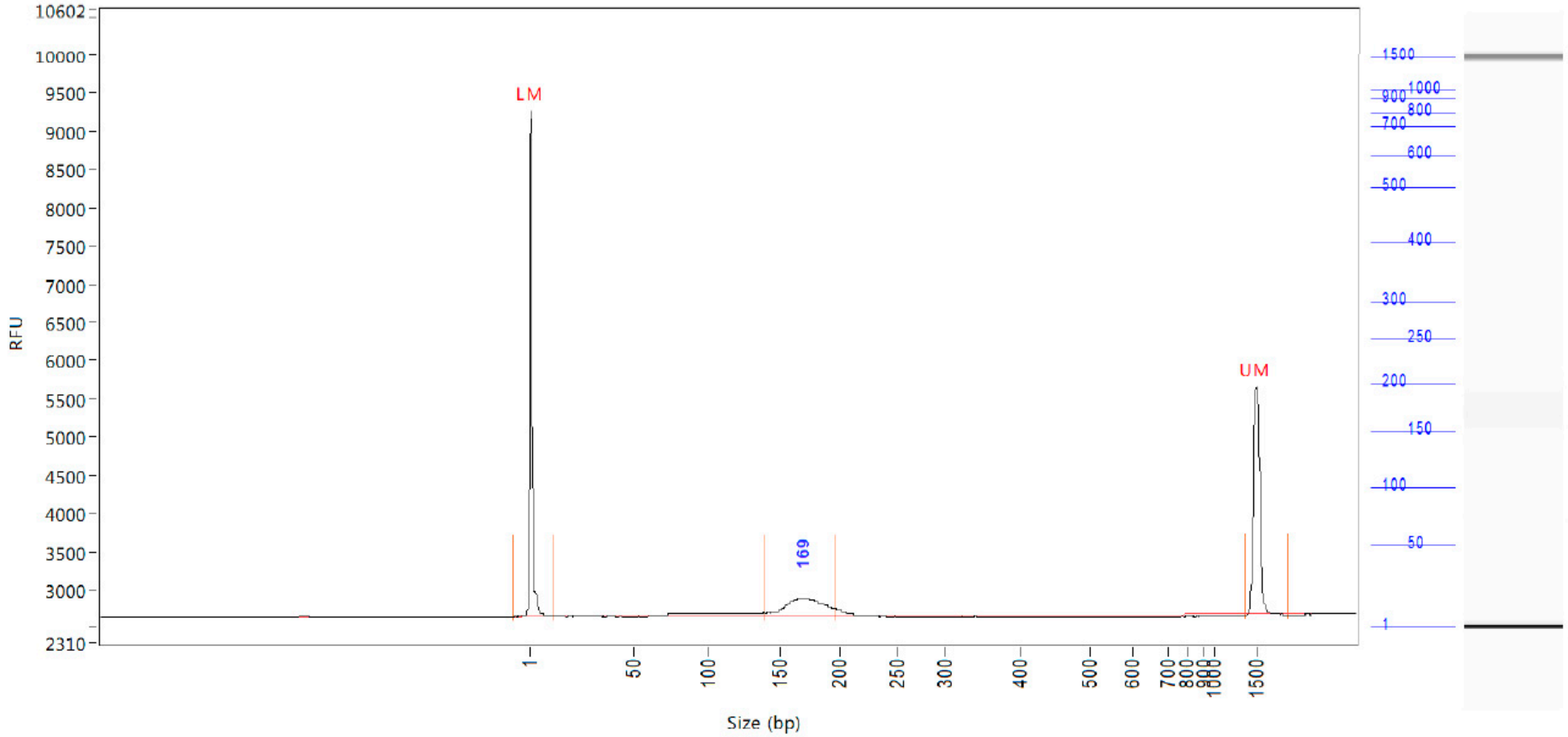

Figure S2: Covariates selection. Heatmap describing the correlations (Pearson's r) between clinical variables and latent surrogate variables (SV1→SV8) extracted from the data

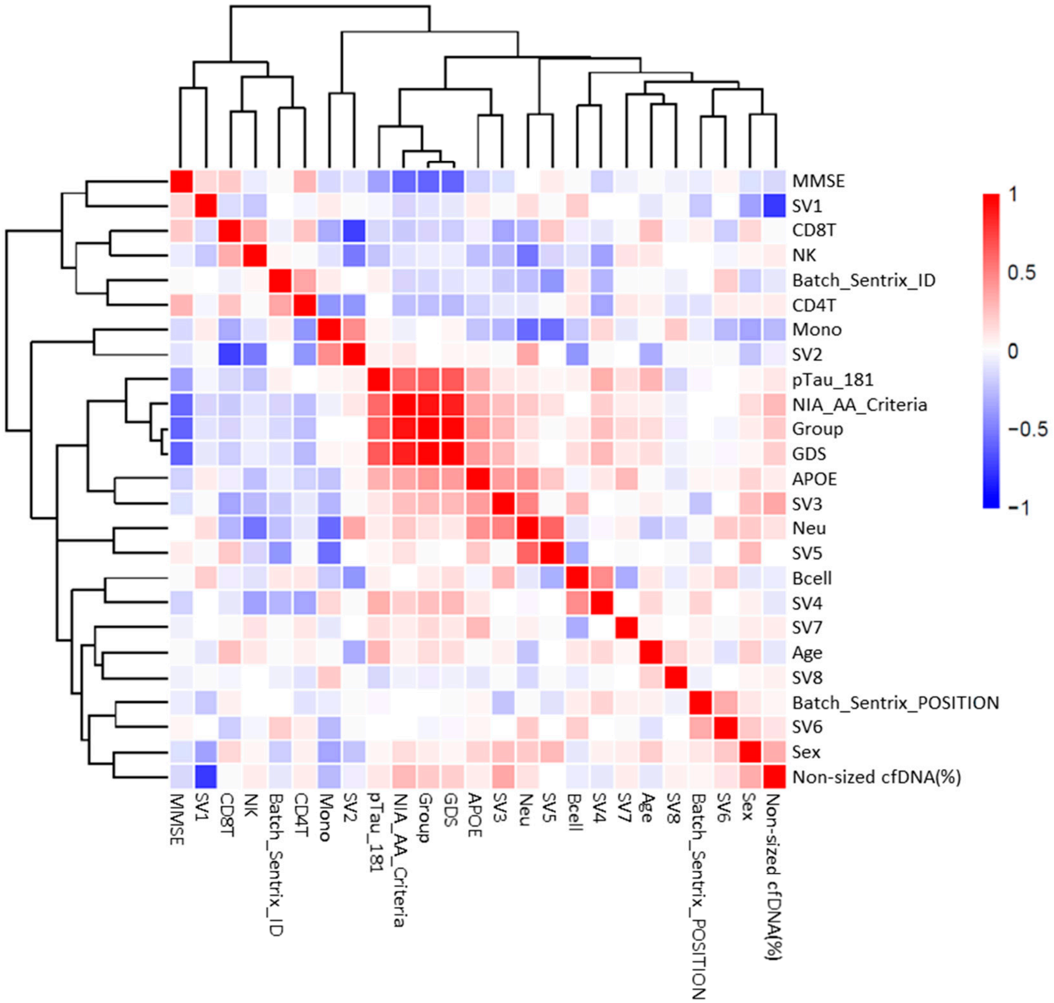

- SV1 → Non-sized cfDNA (%)
- SV2 → Monocytes /CD4+T
- SV3 → APOE/Neutrophils
- SV4 → B-cells
- SV5 → Neutrophils (opp.Monocytes)
- SV6 → Batch (Sentrix Position)
- SV8 → Age
- Genomic DNA α Sex / AD / Neutro

Figure S3: **Characterization of non-sized cfDNA. a)** Example of a sample with the presence of non-sized cfDNA. **(b)** The diagram represents the typical pattern of cfDNA with a peak around 165 bp after performing capillary electrophoresis on a Fragment Analyzer Automated CE System with the DNF-464 High Sensitivity Large Fragment 50Kb Analysis Kit. This kit allows tracking cfDNA fragmentation pattern from 75 bp – 48,500 bp. Presence of genomic DNA is shown from 1,500 – 48,500 bp. bp: base pairs.

a

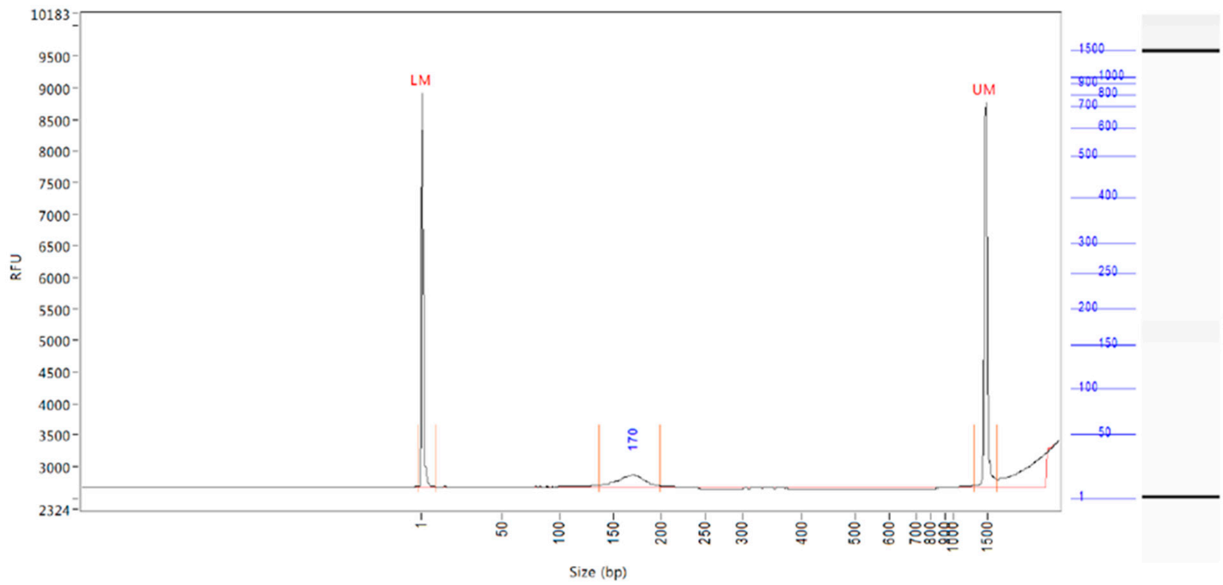

b

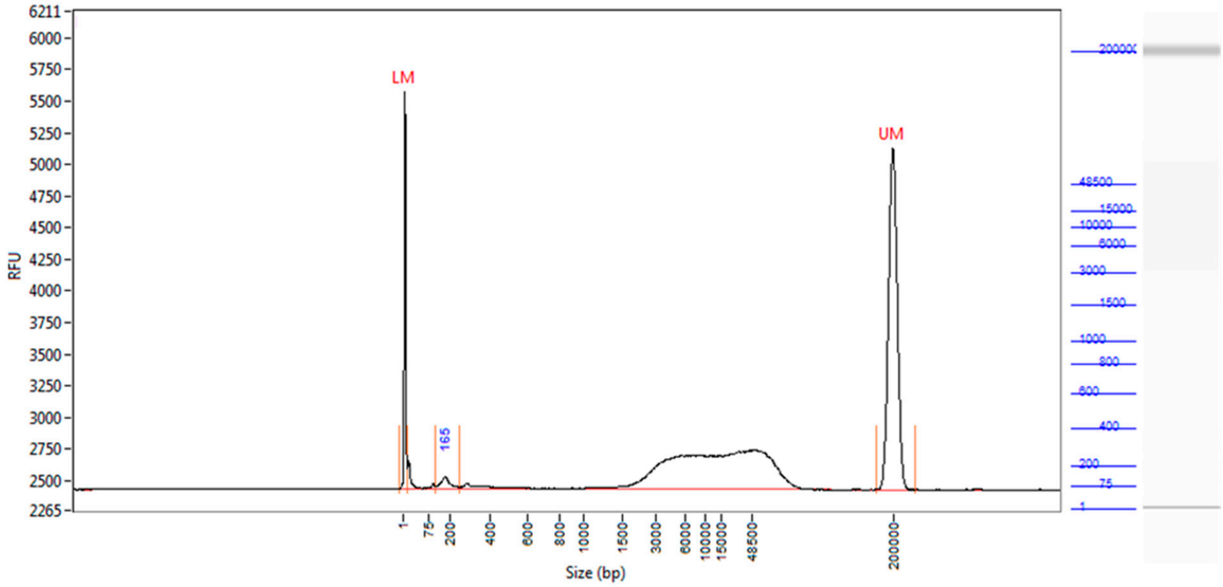

Figure S4: **Box-plot showing the percentage of non-sized cfDNA present in controls and AD patients.** Horizontal lines represent median methylation values and interquartile range for each group.

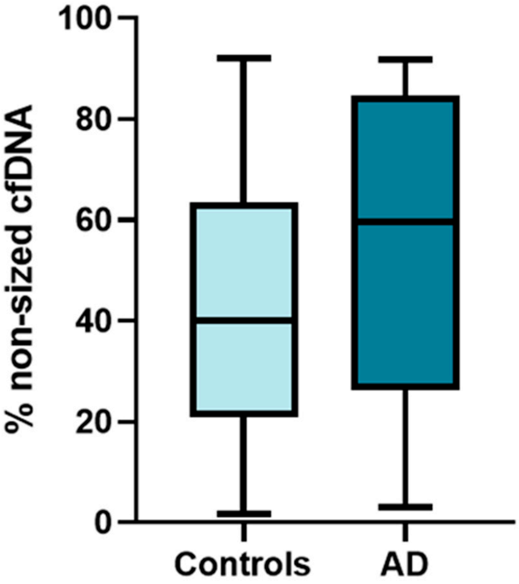

Figure S5: Bioinformatics workflow used in this study: quality control & sample tracking. The diagram shows the bioinformatics pipeline used in this study: procedures for EPIC array methylation data quality control and normalization analysis.

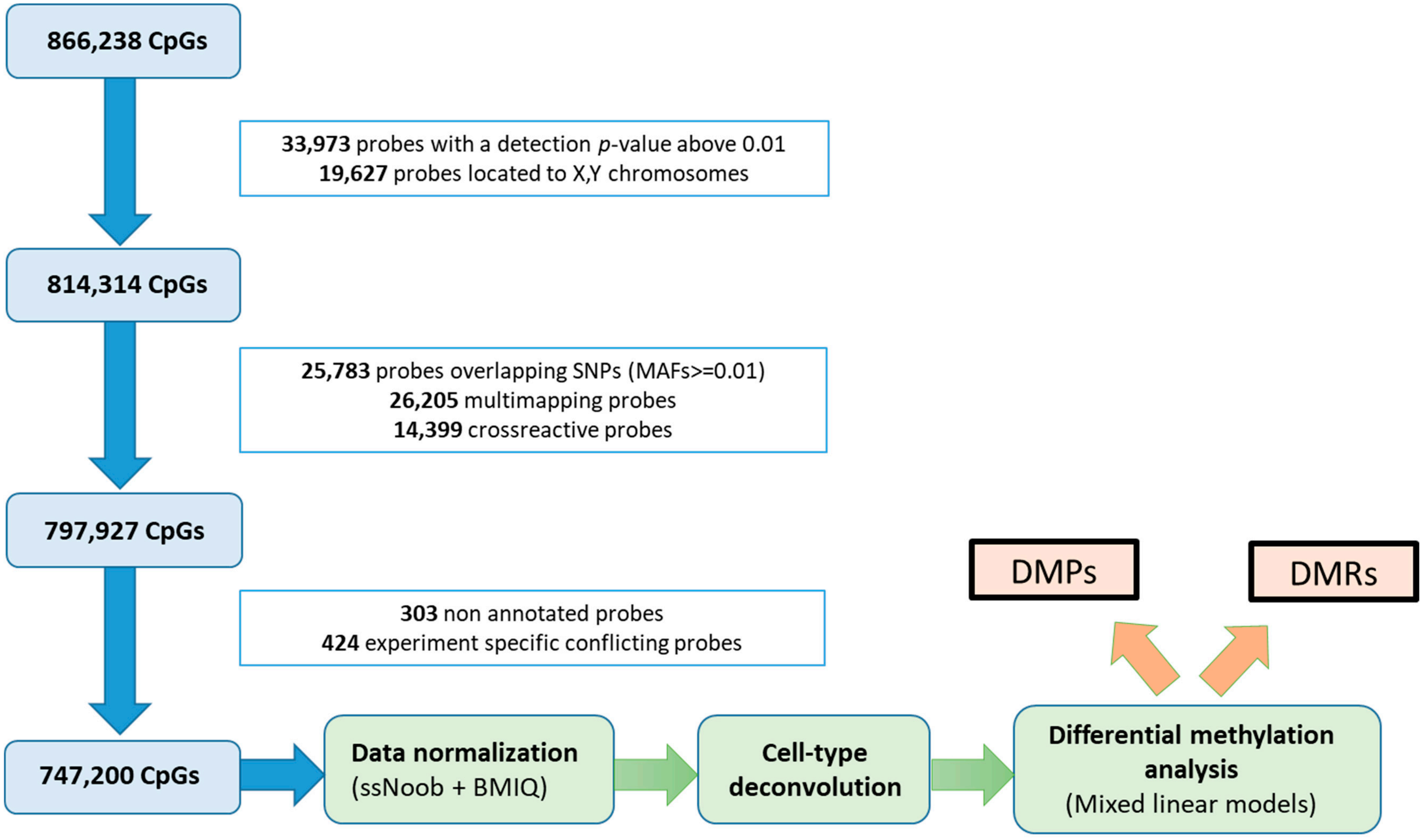

Supplement: Supplementary file 1 [file ijms-26-03419-s001.zip › Supplementary Figures_final.pdf]
